# Inclusion of seasonal variation in river system microbial communities and phototroph activity increases environmental relevance of laboratory chemical persistence tests

**DOI:** 10.1016/j.scitotenv.2020.139070

**Published:** 2020-09-01

**Authors:** Rebecca V. Southwell, Sally L. Hilton, Jonathan M. Pearson, Laurence H. Hand, Gary D. Bending

**Affiliations:** a School of Life Sciences, Gibbet Hill Campus, University of Warwick, Coventry CV4 7AL, UK; b School of Engineering, Library Road, University of Warwick, Coventry CV4 7AL, UK; c Product Safety, Jealott's Hill International Research Centre, Syngenta, Bracknell, Berkshire RG4 6EY, UK

**Keywords:** OECD regulatory tests, Biodegradation, Phototrophs, Temporal variation, Environmental realism

## Abstract

Regulatory tests assess crop protection product environmental fate and toxicity before approval for commercial use. Although globally applied laboratory tests can assess biodegradation, they lack environmental complexity. Microbial communities are subject to temporal and spatial variation, but there is little consideration of these microbial dynamics in the laboratory. Here, we investigated seasonal variation in the microbial composition of water and sediment from a UK river across a two-year time course and determined its effect on the outcome of water-sediment (OECD 308) and water-only (OECD 309) biodegradation tests, using the fungicide isopyrazam. These OECD tests are performed under dark conditions, so test systems incubated under non-UV light:dark cycles were also included to determine the impact on both inoculum characteristics and biodegradation. Isopyrazam degradation was faster when incubated under non-UV light at all collection times in water-sediment microcosms, suggesting that phototrophic communities can metabolise isopyrazam throughout the year. Degradation rate varied seasonally between inoculum collection times only in microcosms incubated in the light, but isopyrazam mineralisation to ^14^CO_2_ varied seasonally under both light and dark conditions, suggesting that heterotrophic communities may also play a role in degradation. Bacterial and phototroph communities varied across time, but there was no clear link between water or sediment microbial composition and variation in degradation rate. During the test period, inoculum microbial community composition changed, particularly in non-UV light incubated microcosms. Overall, we show that regulatory test outcome is not influenced by temporal variation in microbial community structure; however, biodegradation rates from higher tier studies with improved environmental realism, e.g. through addition of non-UV light, may be more variable. These data suggest that standardised OECD tests can provide a conservative estimate of pesticide persistence end points and that additional tests including non-UV light could help bridge the gap between standard tests and field studies.

## Highlights

• Non-UV light increases isopyrazam metabolism, particularly in sediment systems.
• Phototrophs and heterotrophs may both metabolise isopyrazam.
• Seasonal variation in isopyrazam decline only occurred in illuminated microcosms.
• Tests with non-UV light could bridge the gap between laboratory and field studies.

## Graphical abstract

**Figure:**
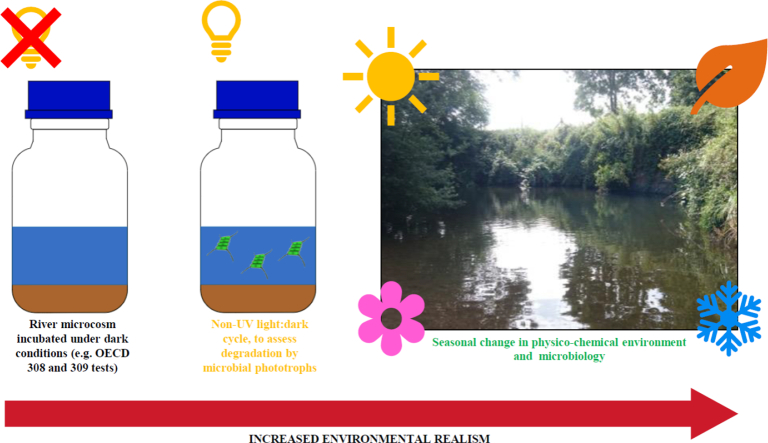

## 1 Introduction

Crop protection products (CPPs) are used widely to improve crop productivity and food security (Hazell, 2002), but they also have the potential to cause detrimental effects to both the environment and human health (Carter, 2000). Prior to being granted approval for use, CPPs are therefore subject to strict regulatory evaluation. The Organisation for Economic Co-operation and Development (OECD) provides a framework for determining the extent to which agrochemicals persist in the environment and the risks they pose to human health and non-target organisms (OECD, 2005).

Microbial degradation is considered to be the most important process determining the environmental fate of CPPs. A number of tests have been developed by the OECD to predict degradation of CPPs and other chemicals in the environment, including OECD test 308, which determines the aerobic and anaerobic transformation of a chemical in aquatic sediment systems (OECD, 2002), and OECD test 309 which determines the aerobic mineralisation of a chemical in surface water (OECD, 2004). These tests provide advantages of consistency, high throughput, and low variability (Carpenter, 1996). Nevertheless, tests are conducted under laboratory conditions which lack environmental realism, and therefore may not give an accurate assessment of the chemical fate and transformation seen in nature (Kowalczyk et al., 2015).

Importantly, OECD tests 308 and 309 are carried out in the dark. Although test 309 has the option for using diffuse light, the guidelines state that dark conditions are preferred and, in test 308, guidelines specifically state that dark conditions should be used to avoid algal blooms (OECD, 2002; OECD, 2004). In the environment, light stimulates growth of phototrophic microorganisms, which use natural sunlight as an energy source (Overmann and Garcia-Pichel, 2006). Phototrophs have been shown to be metabolically capable of chemical biodegradation (Roldán et al., 1998; Lima et al., 2003; Davies et al., 2013a) and several studies have shown that inclusion of non-UV light and the presence of active microbial phototroph communities stimulate CPP biodegradation (Hand and Oliver, 2010; Thomas and Hand, 2012). Evidence from experiments using both soil (Davies et al., 2013a) and water (Thomas and Hand, 2012) suggests that enhanced biodegradation of chemicals under non-UV light could result from a combination of direct degradation by phototrophs and indirect degradation by heterotrophs, which are stimulated by the presence of phototrophs. This degradation mechanism could represent an important transformation pathway which is currently excluded from the OECD test guidelines (OECD, 2002).

OECD test guidelines are ambiguous on when and where sampling of materials from environmental sources should take place, stating that the site should be “selected in accordance with the purpose of the test in any given situation” (OECD, 2002; OECD, 2004). Depending on the CPP used, its mechanism, the target organism, and when a crop is planted, a chemical could be used several times during a cropping season (Caspell et al., 2006; Okonya and Kroschel, 2015) resulting in environmental exposures at several different times of year. Additionally, although CPPs will not necessarily be applied outside of a cropping season, they have the potential to move to different environmental compartments, so may be present in the environment outside of the application time (Tanabe et al., 2001; Vryzas et al., 2009).

Furthermore, the OECD provide limited guidance on microbial characteristics of materials used in tests, specifying that there should be an active microbial population (OECD, 2004), with no consideration of the abundance, diversity, or composition of the community. In the field, degradation rates of chemicals show considerable temporal and spatial variation (Dechesne et al., 2014), with communities also able to adapt to CPP degradation if there has been previous environmental exposure (Pesce et al., 2010). Microbial community composition and functional characteristics can be influenced by a range of physico-chemical parameters including temperature, light intensity, dissolved oxygen, organic matter availability, and nutrient quality and quantity (Kirchman, 2012), so that microbial diversity, including that of microbial phototrophs, vary widely in time and space (Smoot and Findley, 2001; Breuer et al., 2016).

Microbial community composition has major influences on ecosystem function, including chemical biodegradation (Ramakrishnan, 2012). This can arise through a number of factors, including functional and genetic diversity, exchange of genetic elements through horizontal gene transfer, and the nature of microbe-microbe interactions, which can be both synergistic and antagonistic (McGenity et al., 2012; Ramakrishnan, 2012). Generation of false negative biodegradation results in OECD tests has frequently been attributed to variation in the abundance and diversity of microbial communities (Kowalczyk et al., 2015). Importantly, once an inoculum is used in OECD regulatory tests it is treated like a ‘black box’ and the dynamics of microbial communities within test systems are unknown (Kowalczyk et al., 2015). In particular, it is unclear whether microbial characteristics of the starting materials are retained, or whether selection of microbial communities adapted to the test system takes place to generate a defined test system microbiome, irrespective of starting composition.

In this study, we compared degradation of the fungicide isopyrazam in OECD 308 and 309 regulatory test systems in the dark and under non-UV light, to determine the potential importance of microbial phototrophs to biodegradation and mineralisation processes. To investigate the extent to which the outcome of OECD tests is affected by seasonal variation in microbial communities within water and sediment used as inoculum, these tests were repeated 8 times over a 2-year period using materials taken from the same river location. The extent to which differences in degradation rate between collection times and light treatments could be attributed to specific differences in bacterial and microbial phototroph diversity and community composition was also explored. Overall, this study aims to provide insight into the limitations of the current OECD test designs, as well as providing information on key environmental variables which could be included in higher tier studies to improve their environmental realism.

## 2 Materials and methods

**Fig. 1:**
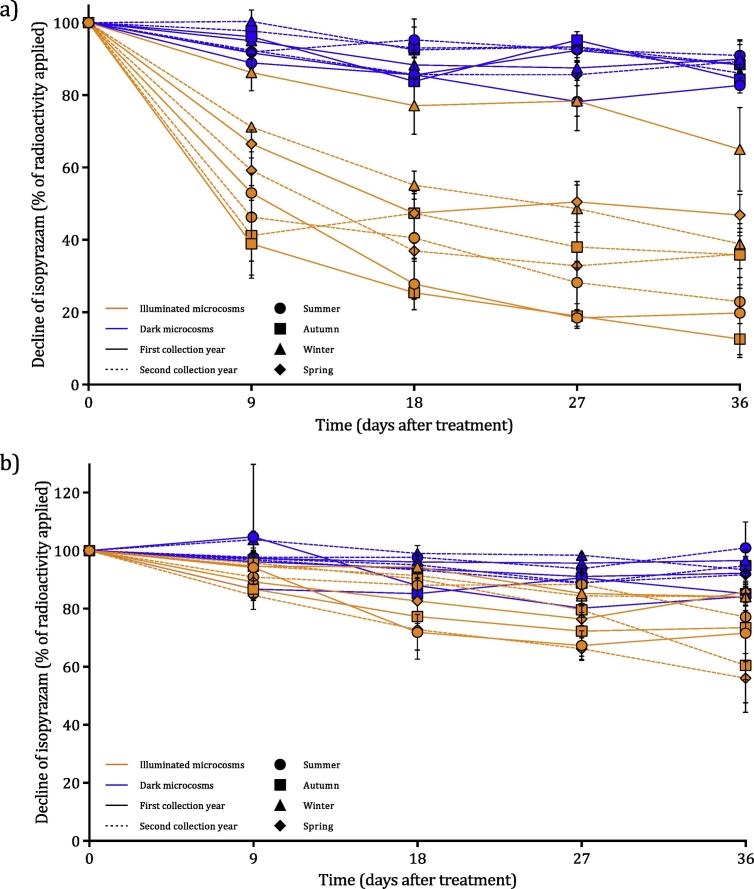
Degradation of isopyrazam in water-sediment microcosms (a) and water-only microcosms (b) as a percentage of the radioactivity originally applied. Degradation of isopyrazam in illuminated (orange) and dark (blue) microcosms over 36 days in summer (circles), autumn (squares), winter (triangles), and spring (diamonds). The first year of each collection time is denoted by a solid line and the second year by a dashed line. Error bars show ± standard deviation.

Environmental inoculum: River water and sediment samples were collected at approximately 3-monthly intervals for 2 years from June 2014. Sample collection was carried out 8 times in total and will be henceforth referred to as “collection times”. Samples were obtained from the River Dene at Wellesbourne, United Kingdom (52°12′02.5″N, 1°36′30.4″W), within the Warwick Crop Centre farm. The sampling site is downstream of a wastewater treatment plant and has a rural catchment area (Supplementary Data (SD); Fig. 1). There is no record of isopyrazam use on the farm and the land bordering the collection site had been set-aside, with no pesticide application since 1995. Analysis of freshly collected water samples using the methods described below showed no detection of isopyrazam at any collection time. At each collection time, sediment and water were collected from the central point in the river, and midway between the central point and each bank, to give triplicate independent samples. Sediment was sandy loam in texture (SD; Table 1) and was sampled within the top 10 cm of the riverbed using a trowel and kept moist with river water. Water was sampled by submerging containers facing upstream. At each collection time, water temperature, light intensity, water depth, water velocity, and water pH were measured. River water samples were filtered through a 106 μm sieve (Fischer Scientific, UK) as detailed in OECD 309 regulatory guidelines (OECD, 2004). Sediment was wet-sieved through a 2.36 mm sieve (Endecotts Ltd., UK) in accordance to OECD 308 regulatory testing (OECD, 2002). Samples were refrigerated at 4 °C and used within 24 h.

**Fig. 2:**
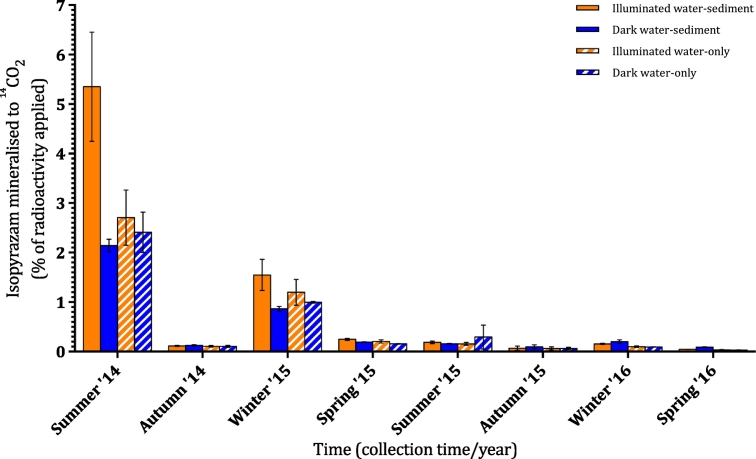
Cumulative amount of isopyrazam mineralised to ^14^CO_2_ as a percentage of the total applied radioactivity. NaOH traps were used to capture mineralised ^14^CO_2_ in illuminated water-sediment (solid orange), dark water-sediment (solid blue), illuminated water-only (dashed orange), and dark water-only (dashed blue) microcosms over 36 days at each collection time. Error bars show ± standard deviation.

Test CPP: Studies were performed using radiolabelled [pyrazole-5-^14^C]-isopyrazam (specific activity 4.736 MBq/mg and 98.6% purity, SD; Fig. 2 and Table 2) supplied by Syngenta, Jealott's Hill International Research Centre, United Kingdom. Isopyrazam was used as a model chemical for this work as it has been shown to be stable in dark incubated water-sediment studies (PPDB, 2017), but is susceptible to phototrophic metabolism (Hand and Oliver, 2010).

Experimental set up: Duran Schott 250 mL clear and amber glass bottles (Scientific Laboratory Supplies, UK) were autoclave sterilised prior to use. Amber bottles were further wrapped in foil to prevent light penetration and the clear and amber bottles were used for illuminated and dark treatments, respectively. At each collection time, the following treatments were set up; dark water-only, illuminated water-only, dark water-sediment, and illuminated water-sediment. In the water-sediment microcosms, 80 mL water and 20 g dry weight equivalent of sediment were added to ensure a 4:1 ratio of water to sediment. In water-only microcosms, 80 mL of water was added. The lid of each bottle was fitted with a crocodile clip with a 20 mL scintillation vial attached so that the vial was suspended inside the bottle. 1 M sodium hydroxide (NaOH, 1 mL) was added to these vials in order to capture any mineralised ^14^CO_2_ from isopyrazam degradation. Microcosms were randomly distributed on a rotary shaker under constant motion at 50 rpm; this sufficiently agitated the water fraction to simulate flow, but did not disturb the sediment fraction. The shaker was in a controlled environment room at 20 ± 2 °C with a 16-hour light and 8-hour dark cycle. Fluorescent 70 W daylight blubs (F70W/865 T8 6 ft., Fusion Lamps, UK) were used with LEE226 filters (Transformation Tubes, UK), which inhibited UV light output. Although isopyrazam is sensitive to aqueous photolysis (EFSA, 2012), it only absorbs light up to 315 nm (Hand and Oliver, 2010); therefore, it can be assumed that any degradation in the illuminated systems was caused by microbial activity, as phototransformation will have been prevented.

Water-sediment microcosms were incubated for 9 days prior to isopyrazam addition to allow communities to equilibrate to laboratory conditions. After 9 days, water was replaced with fresh water collected from the sample site, which had been spiked with [^14^C]-isopyrazam to a concentration of 0.1 mg/L (0.474 MBq/L). This concentration meets with OECD 308 guidelines and ensures that the biodegradation kinetics reflect those expected in the environment (i.e. First-Order kinetics) (OECD, 2004) and is relevant to a worst-case exposure concentration following hypothetical direct overspray of a waterbody (Mohammad and Itoh, 2011). Microcosms were subsequently incubated for up to 36 days.

Destructive harvesting: Destructive harvesting took place at 9, 18, 27, and 36 days after treatment (DAT). At each time point, triplicate microcosms for each treatment were destructively harvested. A nominal 0 DAT value was used, assuming 100% of the applied isopyrazam was in the water fraction. Fresh samples obtained from the river were used for the microbial and water chemistry analyses at 0 DAT. Microbial, water chemistry, and isopyrazam concentration analyses were carried out at all subsequent time points. DNA extraction and microbial profiling were only carried out at 0 and 36 DAT.

Isopyrazam analysis: Residual isopyrazam and ^14^C in the water fraction; The microcosm water fraction was poured gently into a separate storage bottle, so as not to disturb the sediment fraction. As isopyrazam can adsorb to glassware, microcosms were also washed with 8 mL acetonitrile after removal of the sediment fraction (HPLC grade, Fischer Scientific, UK) and the wash collected. Samples of both the water and acetonitrile from each microcosm were analysed by Liquid Scintillation Counting (LSC) using Ecoscint A scintillation cocktail (National Diagnostics, UK) and a Tri-Carb 2800TR scintillation counter (PerkinElmer, US). Water fraction ^14^C content was determined by summing radioactivity determined in the water and acetonitrile wash fractions. The remaining acetonitrile wash was added to the water fraction and the samples sonicated for 3 min using a U300H ultrasonic bath (Ultrawave, UK) in order to lyse any radioactivity sorbed to algal cells. Samples were concentrated by solid phase extraction (SPE) using Oasis HLB SPE cartridges (60 mg, 3 mL, Waters Ltd., UK). To aid retention on the cartridge, samples were acidified using 200 μL concentrated acetic acid (Fischer Scientific, UK) per 60 mL sample. Elution was carried out with methanol (HPLC grade, Fischer Scientific, UK) before evaporation to dryness and resuspension in 1:1 acetonitrile (HPLC grade, Fischer Scientific, UK) and water (HPLC grade, VWR Chemicals, UK). Samples were analysed by High-Performance Liquid Chromatography (HPLC) using a LiChrospher RP-18e μm column (4.0 × 250 mm, Agilent Technologies, US) and a HPLC system (Jasco, UK) connected to a β-RAM radio-HPLC detector (LabLogic, UK). A 1 mL/min flow rate was used with a gradient of 0.2% glacial acetic acid (Fischer Scientific, UK) and acetonitrile (HPLC grade, Fischer Scientific, UK) (SD; Table 3). Chromatograms were quantified using Laura software (version 4, LabLogic, UK). Any additional peaks to isopyrazam on the radio-chromatograms were integrated and the percentages summed to determine metabolite generation.

Residual isopyrazam and ^14^C in the sediment fraction; The sediment fraction was mixed well using a spatula and 10 g dry weight equivalent of sediment from each microcosm was removed. 30 mL 80:20 acetonitrile (HPLC grade, Fischer Scientific, UK) and water (sterile distilled) was added to the sediment and shaken for 1 h at 300 rpm, before centrifugation for 10 min at 228 ×*g*. The supernatant was removed and the pellet subject to the same extraction twice more. Samples of the combined supernatants were analysed using the LSC method above. The sediment extracts were concentrated using nitrogen before re-suspension in 1:1 acetonitrile (HPLC grade, Fischer Scientific, UK) and water (HPLC grade, VWR Chemicals, UK) and HPLC analysis as above.

Solid sediment remaining after extraction was dried and a combustion step carried out to quantify non-extractable residues (NER). An OX500 Biological Oxidizer (R.J. Harvey Instrument Corporation, US) and Oxysolve-C-400 scintillation cocktail (Zinsser Analytic, Germany) were used prior to LSC analysis as above.

Gaseous fraction; The 20 mL scintillation vials containing 1 M NaOH (Fischer Scientific, UK) solution captured ^14^CO_2_ that had been mineralised. These were removed every five days of the experiment and replaced with fresh solutions. Trap solutions were analysed using the LSC method above and the cumulative percentage of applied radioactivity was calculated.

Mass balance: The percentage of radioactivity recovered from each fraction - water, sediment extract, NER in the sediment, and NaOH traps - was summed together for the mass balance.

Water chemistry: Nitrate and phosphate ion concentrations in the water were analysed using NI-14 and PO-14 test kits (Hach, UK), respectively.

Sediment physico-chemical properties: Textural class analysis (percentages of sand, silt, and clay), pH, and percentage of organic carbon were measured by Lancrop Laboratories, Wellington Road, The Industrial Estate, Pocklington, York, United Kingdom. Organic carbon was analysed using a TruMac® CN combustion analyser (LECO Corporation, USA) according to the manufacturer's technical document (LECO Corporation, 2014). pH analysis was performed as described in Agricultural Development and Advisory Service (1986) using an AS300Q Multi Electrode pH Robot (Labfit, Australia). Textural class analysis was performed by a Low Angle Laser Light Scattering technique using a Mastersizer 2000 optical bench (Malvern, UK). Sand was defined as particle sizes between 2 mm to 63 μm, silt between 63 μm to 2 μm, and clay particles under 2 μm.

Microbial analysis: Chlorophyll *a* analysis; Chlorophyll *a* was extracted from both the water and sediment fractions to determine phototrophic organism abundance. Water samples were filtered as in Sartory (1982). A modified version of a method by Ritchie (2006) and described further in Davies et al. (2013b) was used to extract the chlorophyll *a*. Absorbance measurements were carried out using an Ultrospec 1100 pro UV/Visible spectrophotometer (GE Healthcare, UK). Calculations were performed using the formula given in American Public Health Association (1995).

DNA isolation and quantification; Fresh water and sediment taken from the sample site and water and sediment from the microcosms at 36 DAT were analysed. Water samples were filtered on Whatman Anodisc filters (0.2 μm, 47 mm, GE Healthcare, UK) and DNA was isolated both from these and 0.5 g sediment. DNA extraction and quantification were performed as in Álvarez-Martín et al. (2016). Primers were designed so that Nextera XT transposase sequences were added to amplicon primer sets for bacterial (Caporaso et al., 2011) and phototrophic (Sherwood and Presting, 2007) communities. PCR was performed on a GeneAmp PCR System 9700 (Applied Biosystems, US). PCR products were purified and index PCR performed as in Álvarez-Martín et al. (2016), before normalisation using the SequalPrep™ Normalisation Plate Kit (Invitrogen, US), and quantification as above. The final pooled library concentration was 4 nM and this was sequenced using the MiSeq Reagent Kit v3 600-cycle (Illumina, US). Generally, sequence data processing was carried out as in Álvarez-Martín et al. (2016), but for 23S rRNA reads, taxonomy assignment was carried out in ARB (Ludwig et al., 2004) (details in SD; Method 1). Raw sequence data and metadata are stored under the study accession number, SRP132448, in the National Centre for Biotechnology Information (NCBI) Sequence Read Archive (SRA).

Statistical analyses: Statistical analyses were performed using Prism (version 7, GraphPad Software, Inc., US). Significance of differences between treatments for isopyrazam data, water chemistry data, and chlorophyll *a* data were determined using a two-way ANOVA on the entire time course. Two-way ANOVA was also used to evaluate the differences in microbial community relative abundance between microcosm treatments and collection times. One-way ANOVA was performed on sample site variation data and sediment property data. The Tukey method (Haynes, 2013) was used to correct for multiple comparison tests. Alpha (α) diversity was analysed using Fisher's method in the phyloseq package in R (McMurdie and Holmes, 2013), before performing one-way ANOVA as above. Isopyrazam degradation kinetics (DegT50) were estimated using Single First-Order (SFO) kinetics in Computer Aided Kinetic Evaluation (CAKE) (version 3.2, Tessella Ltd., UK). Pearson correlation coefficients were calculated in Prism to determine any links between the variables tested throughout the experiment and both DegT50 and mineralisation data.

## 3 Results

Isopyrazam decline: As in previous studies (Hand and Oliver, 2010), the inclusion of non-UV light had a significant impact on isopyrazam degradation despite OECD tests 308 and 309 being conducted in dark conditions (Fig. 1 and SD; Tables 4 and 5). In particular, degradation in illuminated water-sediment microcosms was significantly quicker compared to all other microcosm treatments, including the illuminated water-only microcosms (generally *p* ≤ 0.0001). Both dark water-sediment and dark water-only microcosms had DegT50s between 101.0 and 2960.0 days and there were no significant differences in degradation rate between the two microcosm treatments, showing that sediment addition only had an impact in illuminated microcosms. It should be noted, however, that these DegT50 values are extrapolated well beyond the study duration. At all collection times, degradation in illuminated water-sediment microcosms (DegT50: 9.0 to 64.4 days) was significantly faster (*p* ≤ 0.01) than dark water-sediment microcosms. In the water-only microcosms, however, the impact of non-UV light was less pronounced (*p* ≤ 0.05) and in illuminated water-only microcosms DegT50s had a broader range of 43.9 to 139.0 days. Indeed, at three collection times (winter 2015, spring 2015, and winter 2016) there were no significant differences between the illuminated and dark water-only microcosm treatments.

In terms of seasonal variation, there was only a clear difference in isopyrazam decline between collection times for the illuminated microcosms and, generally, in the dark microcosms there were no significant differences in isopyrazam degradation rate between collection times (*p* ≥ 0.05); the exception was dark water-only microcosms in the autumn 2014 collection time where residual isopyrazam after 36 days was significantly (85.2%, *p* ≤ 0.05) lower compared to summer 2015 and winter 2016 (101.0 and 93.4%, respectively).

In illuminated microcosms, degradation rate was extremely variable but it was not clearly explained by seasonal differences; in illuminated water-sediment microcosms, there were significant differences between the two collection years in autumn (*p* ≤ 0.0001), winter (*p* ≤ 0.0001), and spring (*p* ≤ 0.05) and, in illuminated water-only microcosms, there were significant differences between the two spring collection times (*p* ≤ 0.0001).

In illuminated water-sediment microcosms, summer and autumn 2014 exhibited the fastest degradation rates and these were significantly (*p* ≤ 0.001) quicker than all of the winter and spring collection times. The degradation rate in winter 2015, on the other hand, was significantly slower (*p* ≤ 0.0001) than all other collection times, with 65.0% isopyrazam still remaining at 36 DAT. The remaining collection times varied between these extremes, and isopyrazam remaining at 36 DAT ranged between 22.9% (summer 2015) and 46.9% (spring 2015). In the illuminated water-only microcosms, degradation was significantly faster in spring 2016 (*p* ≤ 0.05, 56.1%) than all other collection times, apart from summer 2014 and autumn 2015 (71.6 and 60.5%, respectively). Remaining collection times varied between 73.5% (autumn 2014) and 85.8% (summer 2015) isopyrazam remaining at 36 DAT.

**Fig. 3:**
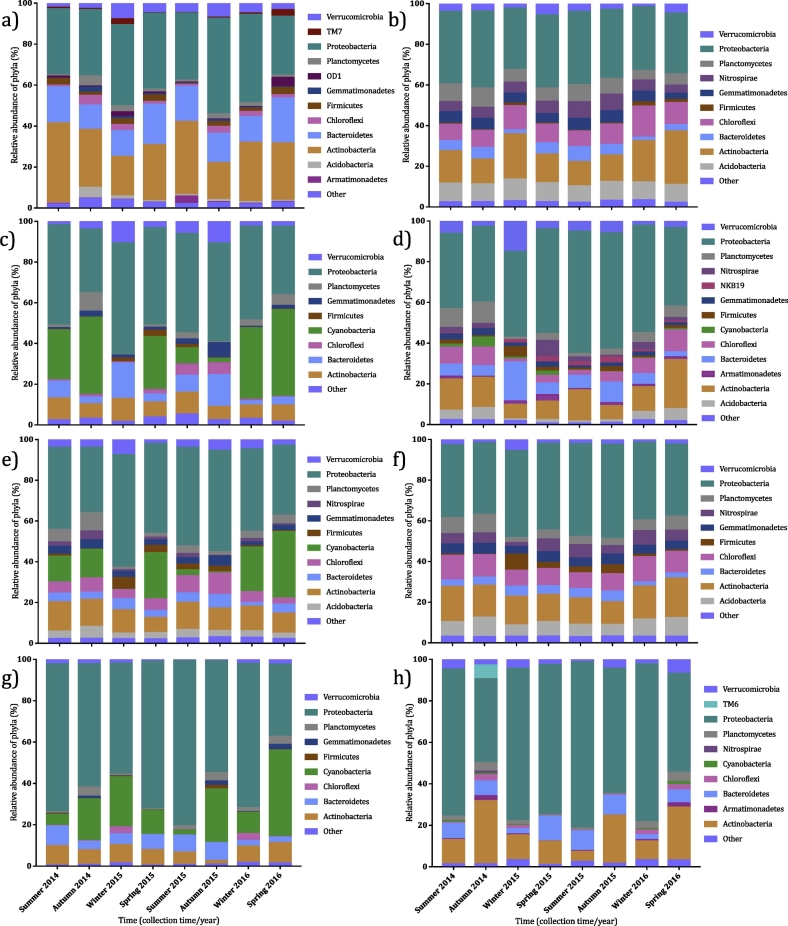
Relative abundance of bacterial phyla between collection times at the sample site and at 36 DAT. Different bacterial phyla are denoted by different colours and phyla comprising <1% of the relative abundance are listed under *other*. Analysis was carried out on fresh water (a) and sediment (b), water in illuminated (c) and dark (d) water-sediment microcosms, sediment in illuminated (e) and dark (f) water-sediment microcosms, and water in illuminated (g) and dark (h) water-only microcosms at 36 DAT.

Metabolite generation: The extent of metabolite generation (SD; Fig. 3) was impacted by the addition of non-UV light, with illuminated water-sediment microcosms producing significantly (*p* ≤ 0.0001) more metabolites compared to the dark water-sediment microcosms. Other than in winter 2015, spring 2015, and winter 2016, illuminated water-only microcosms generated significantly (*p* ≤ 0.05) more metabolites than the respective dark water-only microcosms. There were no significant differences in metabolite detection between the dark water-sediment and dark water-only microcosm treatments, but overall the illuminated water-sediment microcosms produced significantly more metabolites (*p* ≤ 0.0001) compared to the respective illuminated water-only microcosm; this was the case at all collection times, except winter 2015 where there were no significant differences between the two.

In terms of seasonal variation, metabolite formation only varied in the illuminated microcosms and, in the dark microcosms, there was no impact of collection time on the total percentage of metabolites formed, regardless of sediment addition. Full details of variation in the illuminated microcosms are not given here but, briefly, total metabolite formation inversely mirrored the isopyrazam decline data.

**Fig. 4:**
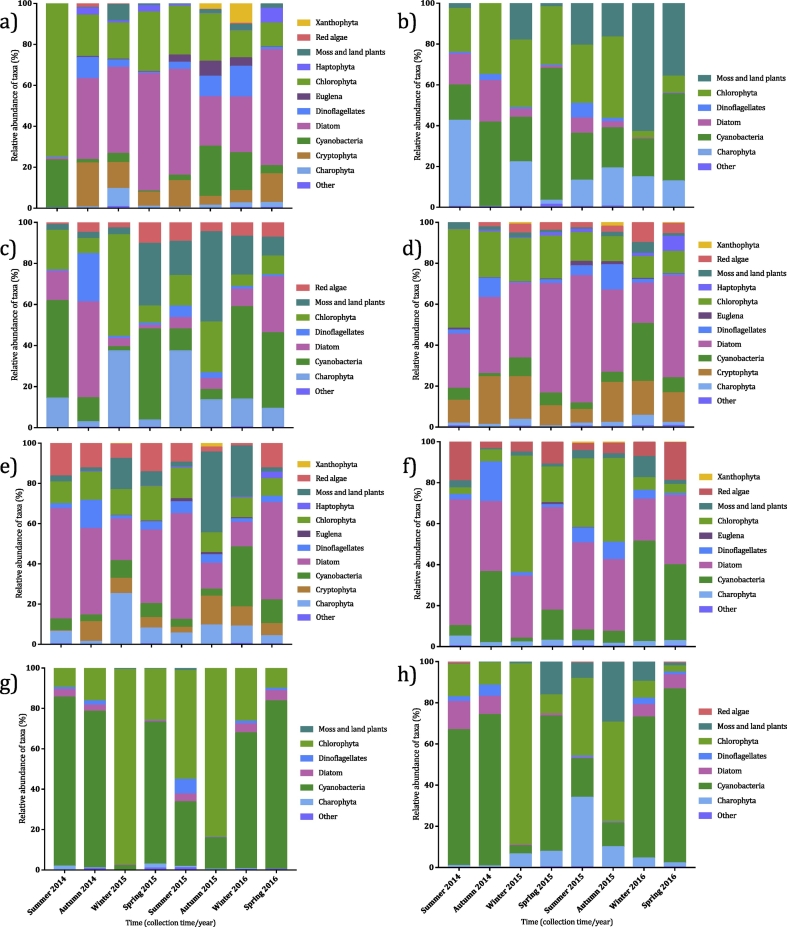
Relative abundance of phototrophic taxa between collection times at the sample site and at 36 DAT. Different phototrophic taxa are denoted by different colours and taxa comprising <1% of the relative abundance are listed under *other*. Analysis was carried out on fresh water (a) and sediment (b), water in illuminated (c) and dark (d) water-sediment microcosms, sediment in illuminated (e) and dark (f) water-sediment microcosms, and water in illuminated (g) and dark (h) water-only microcosms at 36 DAT.

NER: Small amounts of radioactivity were present as NER in the sediment (<7.4%, SD; Fig. 4), however, there were no significant differences between microcosm treatments, regardless of light treatment or collection time (*p* ≤ 0.1326).

Mineralisation to ^14^CO_2_: Mineralisation of isopyrazam to ^14^CO_2_ was highly variable over time (0.03 to 5.4%, Fig. 2). Generally, the addition of non-UV light had no impact on the amount of isopyrazam mineralised. The exception was summer 2014, where illuminated water-sediment microcosms had significantly higher (*p* ≤ 0.0001, 5.4%) mineralisation compared to the other three microcosm treatments (2.1 to 2.7%). There was, however, a significant impact of collection time on total mineralisation (*p* ≤ 0.0001, Fig. 2). This was most pronounced in summer 2014 when mineralisation ranged between 2.1 and 5.4% across microcosms, which was significantly higher compared to all other collection times (*p* ≤ 0.0001). Additionally, in winter 2015, mineralisation ranged between 0.9 and 1.5% across microcosms, which was significantly higher (*p* ≤ 0.01) compared to all other collection times, except summer 2014; this was despite the lower degradation rate of isopyrazam itself in winter 2015. At all other collection times, the total mineralisation was low (<0.5%) for all microcosm treatments.

Correlation between isopyrazam DegT50 and environmental variables: Generally, river depth was greater, water velocity quicker, temperature lower, and light intensity lower at winter collection times relative to summer and autumn collection times, but sediment characteristics did not change over time and there were no significant differences between collection times (SD; Table 1 and Fig. 5). Water temperature at the time of sampling was significantly negatively correlated with the DegT50 data for the illuminated water-sediment microcosms (*r* = −0.80, *p* ≤ 0.0179, SD; Fig. 6). No other environmental variables, including changes in the microbial community, correlated with DegT50 or mineralisation data.

Water chemistry analyses: Generally, NO_3_^−^ concentration (SD; Fig. 7) in the dark water-sediment microcosms was significantly higher (*p* ≤ 0.0001) than the fresh samples and the other microcosm treatments. Although there were fluctuations throughout the experiment, at 36 DAT there were no significant differences between the illuminated water-sediment microcosms and both illuminated and dark water-only microcosm treatments. Overall, PO_4_ concentration (SD; Fig. 8) was not significantly different between microcosm treatments (*p* ≤ 0.4762). Levels did decrease during incubation and, in general, microcosms at 36 DAT contained significantly less PO_4_ compared to the fresh river samples (*p* ≤ 0.0002). In some microcosms there was an exception, for instance in spring 2016, PO_4_ increased in both illuminated and dark water-sediment microcosms compared to the starting sample.

River and microcosm microbial community analyses: In the dark water-sediment microcosms, concentration of chlorophyll *a* remained the same as the starting concentration, while chlorophyll *a* increased during incubation in the illuminated water-sediment microcosms (SD; Fig. 9), with significantly more developing in summer and autumn 2014 (*p* ≤ 0.0001) compared to the other collection times. Additionally, there was a higher chlorophyll *a* concentration in the illuminated water-sediment microcosms compared to the illuminated water-only microcosms (*p* ≤ 0.0001). During incubation, there were increases in chlorophyll *a* concentration in illuminated water-only microcosms at several time points, but the timing of this was variable, and the increase did not persist until the end of the incubation period.

Freshly collected water and sediment supported significantly different bacterial communities and, within each inoculum type, composition varied between collection times (*p* ≤ 0.0001). Generally, sediment communities were stable over time, and had larger proportions of Acidobacteria, Chloroflexi, Gemmatimonadetes, and Nitrospirae than the water fraction (Fig. 3). In the water, communities were dominated by Proteobacteria, Actinobacteria, and Bacteroidetes, although overall composition varied markedly over time (*p* ≤ 0.0001). Following incubation, sediment bacterial communities from the dark water-sediment treatments showed relatively little change to the starting composition, but in all other microcosms and inocula there were substantive changes in community composition following incubation; this varied according to both microcosm and collection time. Generally, incubation resulted in increased relative abundance of Proteobacteria across all microcosms. In illuminated microcosms, there was generally an increase in Cyanobacteria relative abundance, but this was variable (0.04 to 42.62%), with winter 2015 illuminated water-sediment microcosms in particular having a low relative abundance in both the water and sediment fractions (0.04 and 0.07%, respectively).

Freshly collected water and sediment supported significantly (*p* ≤ 0.0001) different phototroph communities (Fig. 4) and, within each inoculum, composition varied significantly (*p* ≤ 0.0001) between collection times. Freshly collected water had a higher relative abundance of Diatoms and Cryptophyta than sediment, which generally supported a higher relative abundance of Charophyta and Moss and Land Plants. The water in the dark water-sediment microcosms generally maintained a similar phototroph profile to the starting material. All other water and sediment inocula showed marked changes in composition following incubation. In illuminated water-sediment microcosms, there was generally an increased relative abundance of Charophyta and Red Algae, and the increased relative abundance of Red Algae was also seen in the sediment fraction of dark water-sediment microcosms. Cyanobacteria relative abundance increased in both the illuminated and dark water-only microcosms, with the exception of the winter 2015 samples, mirroring findings in the bacterial community analysis.

Both bacterial and phototrophic α diversity (SD; Figs. 10 and 11) was generally higher (*p* ≤ 0.01) in fresh water samples compared to microcosms at 36 DAT. For the sediment, however, fresh samples only had a significantly higher α diversity compared to the sediment in illuminated microcosms (*p* ≤ 0.0001) and there was typically little difference between the fresh sediment and sediment in the dark microcosms. For bacteria, dark water-sediment microcosms generally had higher α diversities compared to the other microcosms at 36 DAT in both the water and the sediment fractions (*p* ≤ 0.0001).

## 4 Discussion

The inclusion of non-UV light-dark cycles in OECD 308 and 309 studies using river inoculum increased the degradation rate of isopyrazam relative to test systems maintained in continuous darkness (in which there was little degradation). Similar findings were generated in studies using water and sediment sourced from a lake (Hand and Oliver, 2010; Hand and Moreland, 2014), therefore confirming these results are not specific to lake water and sediment systems. This increased degradation rate was more pronounced in illuminated microcosms containing sediment, with sediment addition having no significant impact on degradation in the dark.

The impact of seasonal variation has not previously been studied in regard to OECD test outcome. When the studies were conducted in continuous darkness, there was no significant variation in DegT50 from season to season, with or without sediment addition. This was unsurprising given how little degradation was observed under dark conditions. There was, however, substantial seasonal variation in isopyrazam degradation rate in the illuminated microcosms. Again, this was more pronounced in the systems which contained sediment, but it was not predictable, i.e. degradation did not follow a simple seasonal pattern. It was noted, however, that variation in illuminated water-sediment DegT50 correlated with the temperature of the river water at the time of sampling. In the absence of sediment, the overall degradation rate was slower and, on occasions, not statistically significantly faster than the degradation rate in dark systems; therefore, the seasonal variation was also less significant.

One unexpected observation was the seasonal variation in the extent of isopyrazam mineralisation to ^14^CO_2_. Generally, mineralisation was negligible (<0.5%) which was in line with the findings of the regulatory study package (Syngenta, unpublished data), however, on two occasions (summer 2014 and winter 2015) mineralisation reached a maximum of 5.4% applied radioactivity. This seemed to be unrelated to the inclusion of light-dark cycles or sediment, as mineralisation was significantly higher at these collection times under all conditions. Furthermore, there was no clear relationship between the mineralisation rate and the DegT50 of isopyrazam itself; for example, the isopyrazam DegT50 in the winter 2015 collection time was the slowest among the illuminated water-sediment microcosms, yet this was one of the collection times which showed high amounts of mineralisation. This would tend to suggest that a different microbial population is responsible for the initial step of parent compound degradation than the subsequent steps which result in mineralisation.

Chlorophyll *a* data clearly demonstrates that there was an increased abundance of phototrophs in the illuminated microcosms, suggesting that phototrophic communities likely played a role in the increased degradation of isopyrazam, as has been described in multiple studies with a range of chemicals (Roldán et al., 1998; Lima et al., 2003; Thomas and Hand, 2011; Davies et al., 2013a), including specifically isopyrazam transformation (Hand and Oliver, 2010; Hand and Moreland, 2014). These data suggest that phototrophs resident in both the water column and within the sediment contributed to biodegradation. This could occur via a number of mechanisms. Phototrophs, particularly cyanobacteria, have been shown to directly metabolise a range of chemicals, while carbon fixation by phototrophs could also stimulate the biomass of heterotrophs, thereby increasing degradation rate (Davies et al., 2013a). Furthermore, photosynthesis is associated with the formation of reactive oxygen species (Foyer, 2018), which could indirectly contribute to degradation.

Both bacterial and phototroph community composition varied in sediment and water across collection times; however, no clear links between the changes in bacteria or phototroph phyla composition and the enhanced degradation rate could be established. Cyanobacteria were the most consistent group to show an increase in relative abundance in illuminated treatments. At the end of the winter 2015 collection time, Cyanobacteria were present in very low abundance relative to the other collection points in illuminated water-sediment systems and isopyrazam degradation was also slower compared to the other collection times. Nevertheless, overall Cyanobacteria relative abundance did not correlate with DegT50, as at some collection times isopyrazam decline was still fast despite relatively low Cyanobacteria abundance (e.g. summer and autumn 2015). Charophyta relative abundance also increased in illuminated water-sediment microcosms compared to the other treatments, but similarly this was not directly correlated with DegT50. Additionally, Cyanobacteria were still present in the dark microcosms, in which isopyrazam degradation was consistently slow, although phototrophs in the dark systems are likely to be dormant.

As there were no clear links between specific microbial taxa and degradation or mineralisation rates, a clear identification of the microbial species responsible for isopyrazam metabolism was not possible; however, these results suggest that the microbial species capable of isopyrazam metabolism were present at each collection time, and that either phototrophic taxa which contribute to degradation varied over time, or that the metabolic capability of specific microbial phototrophs differed from season to season. Mineralisation was significantly higher in summer 2014 and winter 2015, suggesting species better adapted at performing the later metabolic steps were present or enhanced at these collection times. Additionally, since mineralisation also occurred in the dark, heterotrophic microbes clearly play a role in isopyrazam transformation alongside phototrophic communities.

Bacterial and phototroph communities in river water and sediment varied between collection times and microbial composition changed during incubation. The microbial composition following 36 days of incubation was variable across the collection times, despite incubation under defined conditions. Within the environment, physico-chemical variables are important determinants of microbial abundance and composition (Luo et al., 2019) and different microbial taxa dominate at different times of year (Crump et al., 2009). Species can acclimatise to new environments (Bárcenas-Moreno et al., 2009), and the transfer from the environment to controlled laboratory conditions in this study may have required an acclimatisation period. This could include phenotypic and genotypic sorting to select communities better adapted to the laboratory conditions, such as temperature or aeration status (Bárcenas-Moreno et al., 2009). In terms of temperature changes, community alteration could depend on the extent of the temperature shift and whether it is within the historical range of the environment (Kuffner et al., 2012). Data collected by the Environment Agency (2017) between 2010 and 2017 show that water temperature of the River Dene ranges from 1.0 °C (January 2010) to 18.6 °C (July 2013), indicating that the 20 °C used in the laboratory is at the limit of normal environmental exposure. Relative to the field site at the time of collection, water temperatures were 4 °C higher in the laboratory in summer 2014, but in winter 2015 they were 13.5 °C higher in the laboratory than in the environment. Interestingly, there was a negative correlation between the DegT50 in illuminated water-sediment microcosms and the water temperature at the sampling site at the time of collection, which could indicate that acclimatisation plays an important role in determining functional attributes of communities which grow in laboratory incubation conditions. If the microbial communities were better acclimatised to the new laboratory conditions, isopyrazam transformation functionality and the metabolic competence of these communities may have been greater.

The dark treatment microcosms used in this study most closely represented the OECD regulatory tests, and degradation was significantly slower in these systems compared to the illuminated treatments. Although it is possible that longer incubation times could have resulted in changes to the degradation kinetics, and potentially higher degradation rates, this has previously only been noted in situations when communities have had prior exposure to the chemical, and adaptation can take place (Pesce et al., 2013). Microbial populations are exposed to light in surface water and, depending on the depth of the water column, also in the sediment; thus, phototrophic populations are active components of environmental communities. When non-UV light was added to the experimental design in the laboratory, DegT50s were much shorter than those predicted in the standardised OECD tests. This suggests that regulatory tests are conservative and underestimate degradation rates and, therefore, the persistence end points that the DegT50 values are ultimately used for. Nevertheless, following incubation, microbial populations in the dark treatment most closely resembled those in the starting material, with substantive changes in community composition taking place in the light. This could reflect lack of microbial growth within dark incubated systems, although changes to the NO_3_^−^ and PO_4_ concentrations suggest that microbial populations were active. The degradation of all compounds will not necessarily be enhanced by non-UV light as transformation processes are compound specific (Davies et al., 2013a), but the inclusion of non-UV light can give information on whether phototrophic communities, which are excluded using the test guidelines (OECD, 2002), could play a potential role in compound metabolism.

In general, seasonal variation was more prominent in the illuminated systems, so the inclusion of light in studies could lead to ambiguities, as DegT50s were not consistent among the different collection times. Nevertheless, it is important that the contribution of all potential metabolising taxa is considered when assessing the persistence of any chemical that is released into the environment; additional studies including non-UV light could help bridge the gap between discrepancies between standardised OECD tests, which look at transformation processes in isolation, and behaviour under natural conditions or in field studies (Beulke et al., 2000). Seasonal variation was likely due to the specific microbial consortia present at any given collection time and studies have suggested that this is the main driver for inconsistencies in biotransformation estimations (Thouand et al., 1995). Additionally, we demonstrated significant loss of water and sediment microbial biodiversity in both illuminated and dark incubated small-scale OECD tests and this could also impact the extent to which biodegradation rates reflect those experienced in the environment (Kowalczyk et al., 2015). If the degraders from the environment are not active in the microcosms, as is the case in dark OECD tests excluding phototrophs responsible for isopyrazam metabolism, the regulatory guidelines will be unable to adequately predict realistic transformation rates.

## 5 Conclusions

This study focused on the addition of non-UV light to OECD 308 and 309 test systems and compared these to the dark conditions normally used in these tests. Isopyrazam degradation was faster when incubated under non-UV light, particularly in the water-sediment microcosm treatments, suggesting that phototrophic communities played a role in metabolism. It is likely that heterotrophic communities also played a role, as increased mineralisation to ^14^CO_2_ at certain collection times was not specific to the illuminated treatments. Although further work with other compounds would be required, these data suggest that additional tests under non-UV light could help bridge the gap between standard tests and more realistic field studies.

Studies were carried out eight times over two years and seasonal variation in isopyrazam decline only occurred in the illuminated treatments. Although microbial community composition altered both seasonally and during incubation, these changes were not clearly linked to the potential for isopyrazam degradation. Results from the illuminated treatments demonstrated that isopyrazam degraders were present throughout the year but with varying capabilities, highlighting the need to ensure that CPP metabolisers are sufficiently represented in the OECD test inoculum.

Lastly, in the illuminated water-sediment microcosms, degradation was negatively correlated to the river temperature at the time of collection. Additional work would be required to determine whether the microbial communities are genuinely less active depending on river temperature at certain times of the year, or whether this is due to the ability for microbial communities to acclimatise to the temperature used in regulatory tests.

## CRediT authorship contribution statement

**Rebecca V. Southwell:** Methodology, Formal analysis, Writing - original draft, Writing - review & editing. **Sally L. Hilton:** Formal analysis, Writing - review & editing. **Jonathan M. Pearson:** Conceptualization, Methodology, Formal analysis, Writing - review & editing. **Laurence H. Hand:** Conceptualization, Methodology, Formal analysis, Writing - original draft, Writing - review & editing. **Gary D. Bending:** Conceptualization, Methodology, Formal analysis, Writing - original draft, Writing - review & editing.

## Declaration of competing interest

The authors declare that they have no known competing financial interests or personal relationships that could have appeared to influence the work reported in this paper.

## Appendix A Supplementary data

Supplementary material Image 1
