# Supplementary material for: Inclusion of seasonal variation in river system microbial communities and phototroph activity increases environmental relevance of laboratory chemical persistence tests
Source: Sci Total Environ. 2020 Sep 1;733:139070. doi: 10.1016/j.scitotenv.2020.139070 (PMC7298614; doi:10.1016/j.scitotenv.2020.139070)
Supplement: Supplementary file 1 — Supplementary material [file mmc1.pdf]

Supplementary data

Inclusion of seasonal variation in river system microbial communities and phototroph activity increases environmental relevance of laboratory chemical persistence tests

Rebecca V. Southwell [*,a,c], Sally L. Hilton [a], Jonathan M. Pearson [b], Laurence H. Hand [c], Gary D. Bending [a]

[a] School of Life Sciences, Gibbet Hill Campus, University of Warwick, Coventry, CV4 7AL, U.K.

[b] School of Engineering, Library Road, University of Warwick, Coventry, CV4 7AL, U.K.

[c] Product Safety, Jealott's Hill International Research Centre, Syngenta, Bracknell, Berkshire, RG4 6EY, U.K.

[*] Corresponding author, e-mail: rebecca.v.southwell@hotmail.com.

Number of figures: Eleven.

Number of tables: Five.

Number of methods: One.

All references associated with the supplementary data are listed at the end of this document.

Figures

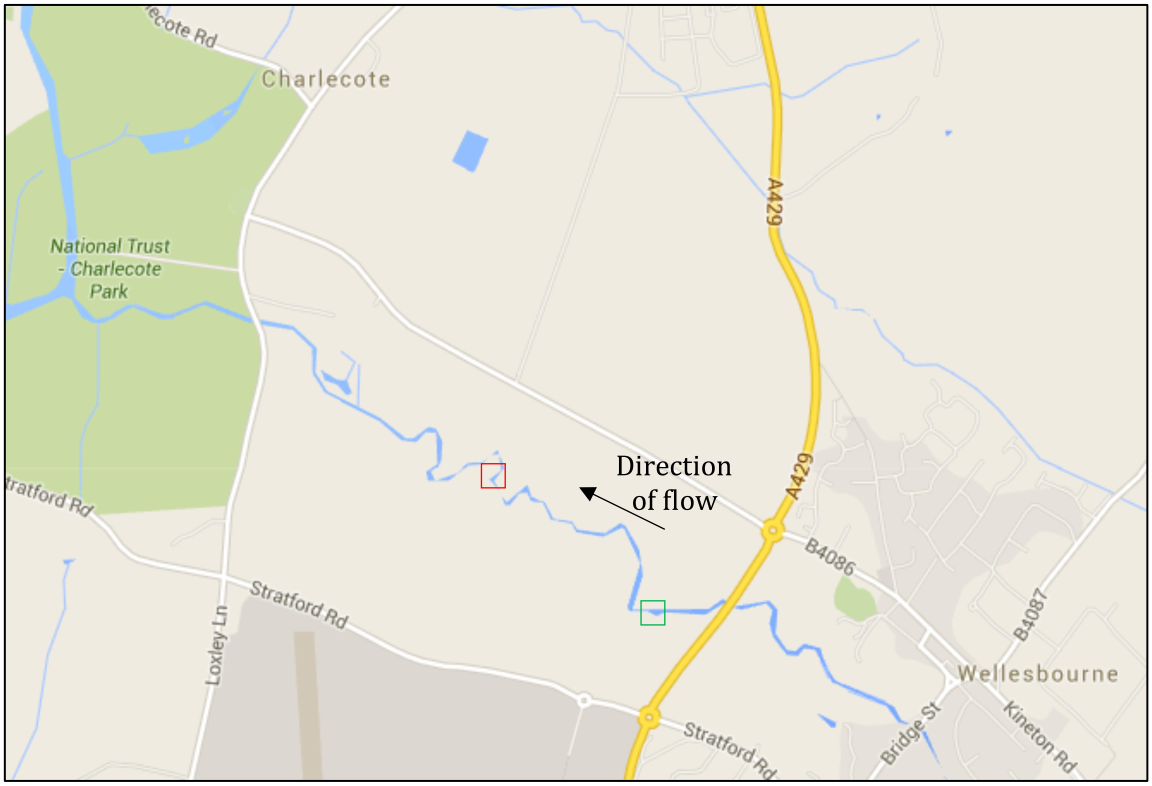

Figure 1. Map showing the River Dene, Wellesbourne, United Kingdom. The red square denotes the location of the sample site downstream of a wastewater treatment plant, which is shown by the green square (Google, 2016).

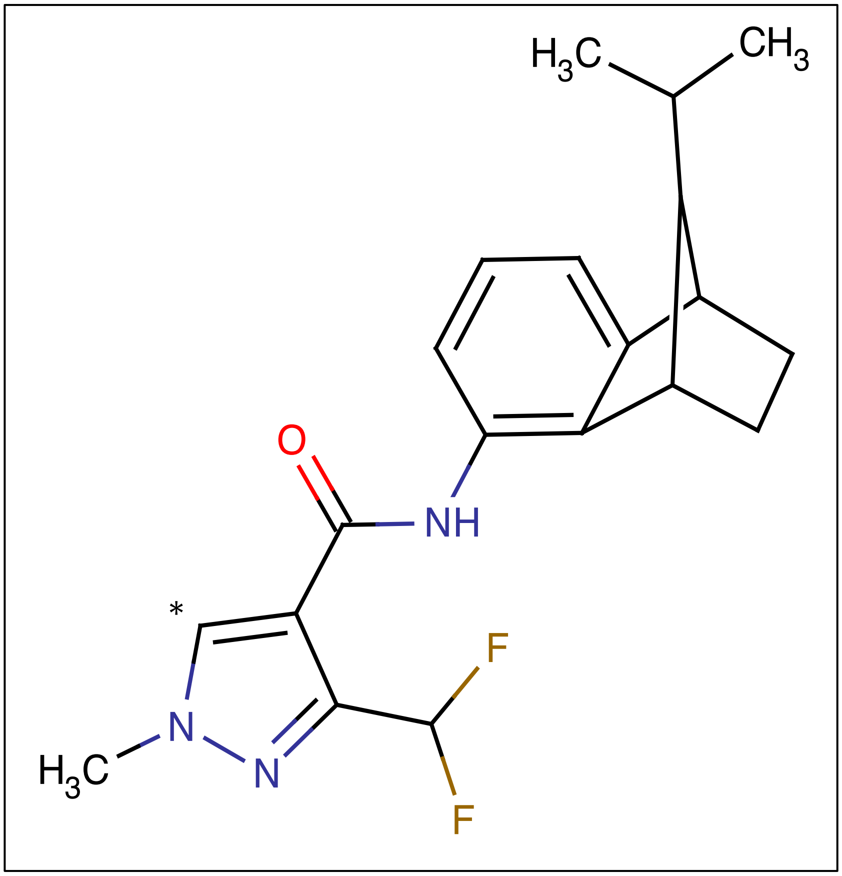

Figure 2. Structure of isopyrazam. The * denotes the position of the radiolabelling and the mixture was made up of 89.7 % *syn*-epimer and 9.7 % *anti*-epimer. Created using ChemDraw (PerkinElmer, US).

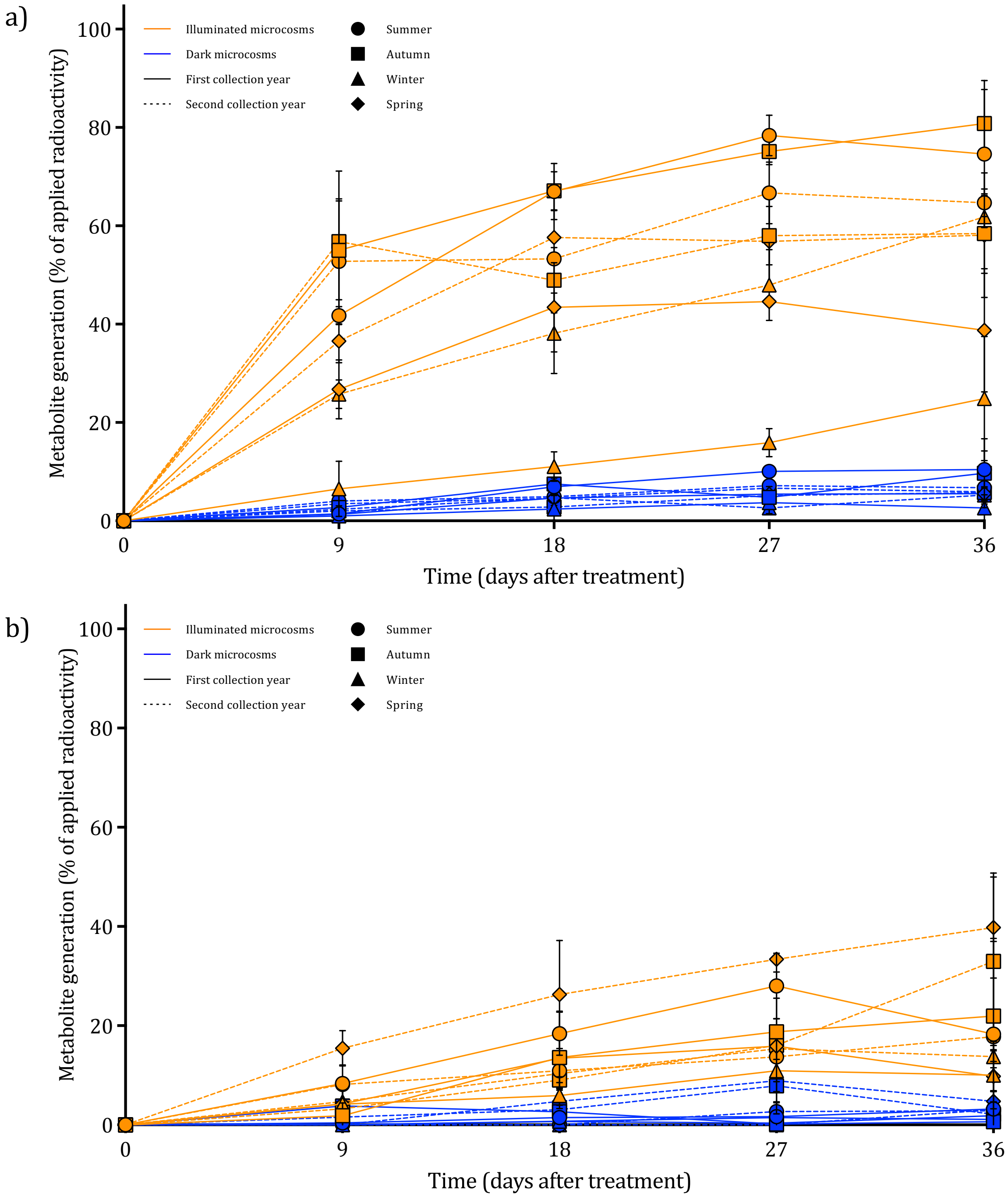

Figure 3. Generation of metabolites in water-sediment (a) and water-only (b) microcosms as a percentage of the radioactivity originally applied. Generation of metabolites in illuminated (orange) and dark (blue) microcosms over 36 days in summer (circles), autumn (squares), winter (triangles), and spring (diamonds). The first year of each collection time was

denoted by a solid line and the second year by a dashed line. Error bars show ± standard deviation.

a)

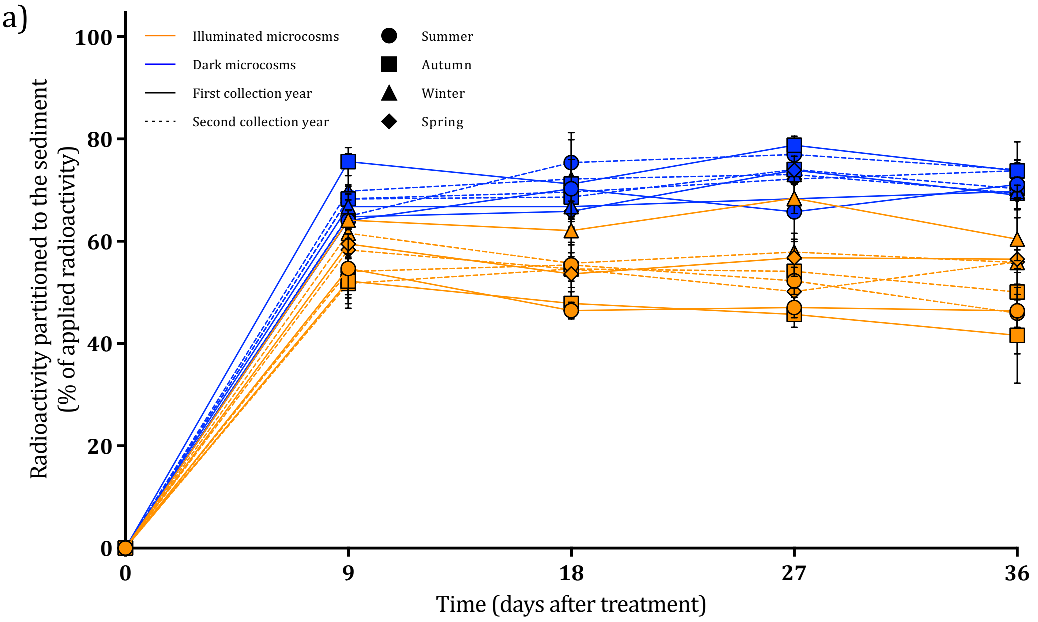

b)

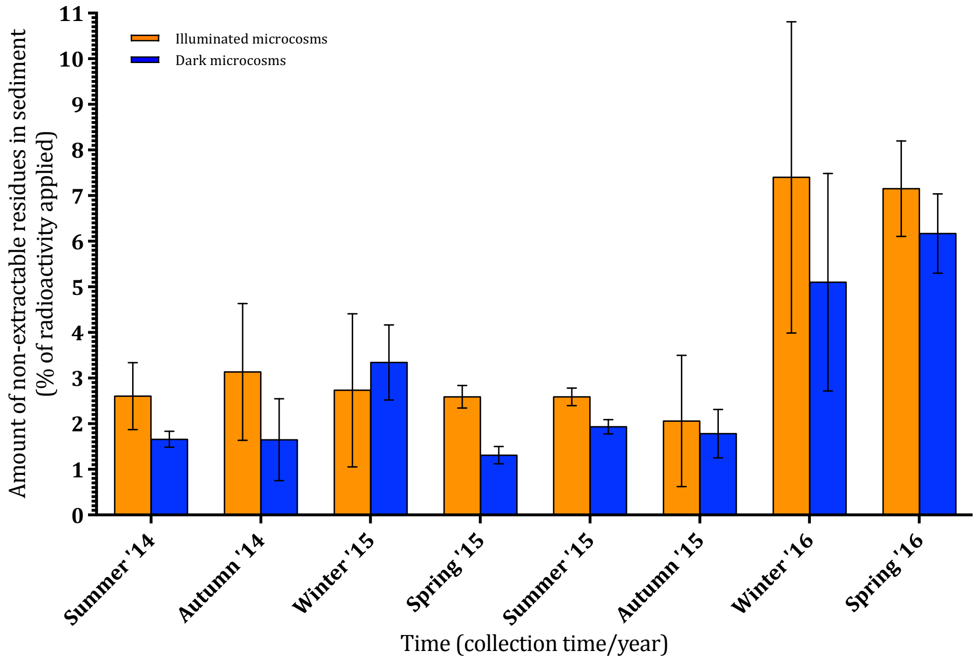

Figure 4. Partitioning of radioactivity to the sediment fraction (a) and amount of non-extractable residues in the sediment at 36 DAT as a percentage of the total applied radioactivity. Sediment partitioning of radioactivity in illuminated (orange) and dark (blue) water-sediment microcosms over 36 days in summer (circles), autumn (squares), winter

(triangles), and spring (diamonds). The first year of each collection time was denoted by a solid line and the second year by a dashed line (a). Non-extractable residues in the sediment fraction from illuminated (solid orange) and dark (solid blue) water-sediment microcosms at 36 DAT (b). Error bars show ± standard deviation.

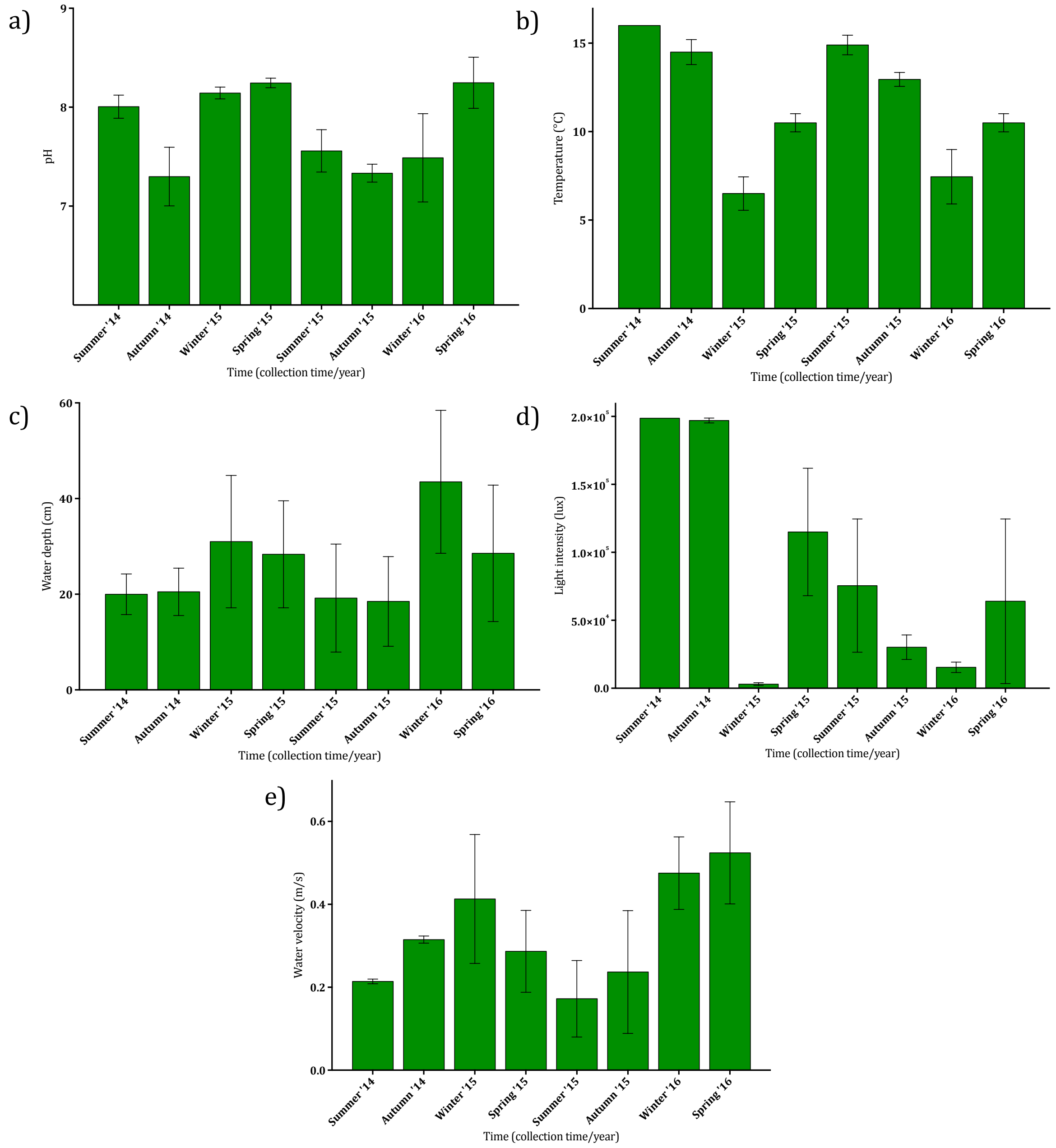

Figure 5. Variation in conditions at the sample site between collection times. Variation at the sample site of pH (a), water temperature (b), water depth (c), light intensity (d), and water velocity (e). Error bars show ± standard deviation.

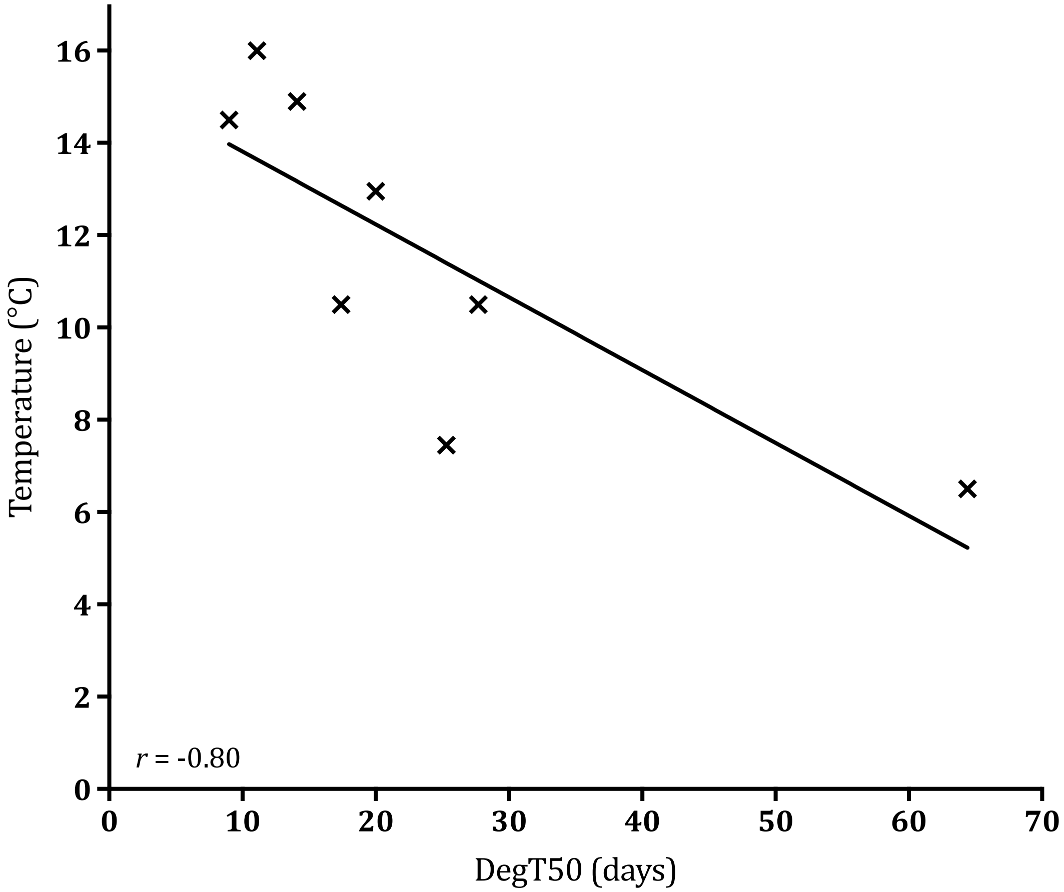

Figure 6. Correlation between illuminated water-sediment microcosm DegT50 values and water temperature at the sample site. Data points show average DegT50 values from each collection time. The Pearson correlation coefficient was -0.80.

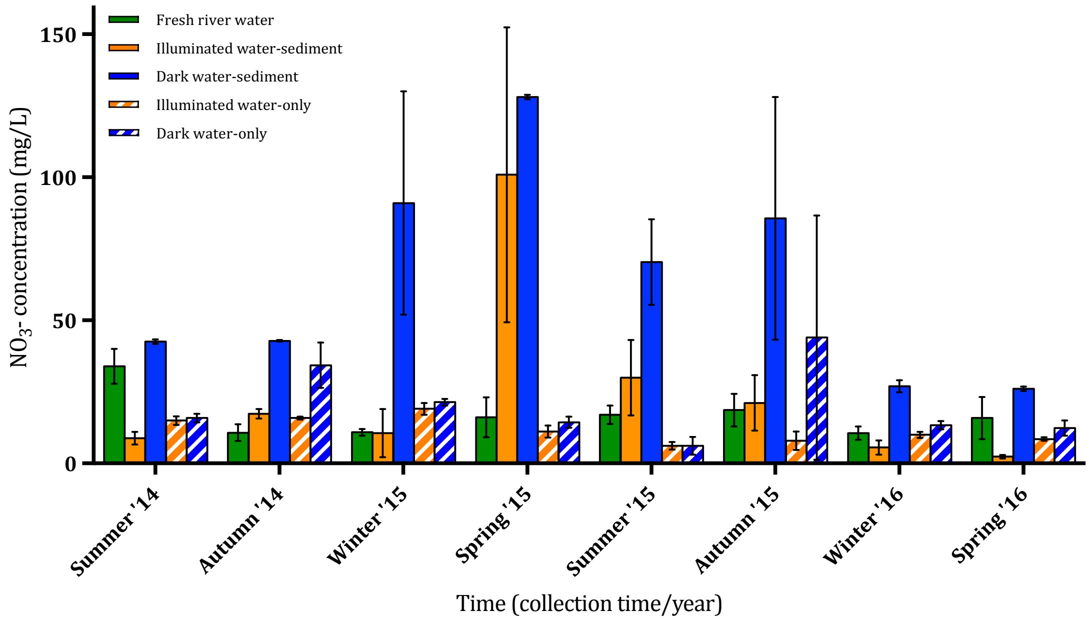

Figure 7. Water NO_3- concentration of the fresh samples and at the end of each collection time. NO_3- concentration in the water was quantified for the fresh samples from the river (solid green) and in the illuminated water-sediment (solid orange), dark water-sediment (solid blue), illuminated water-only (dashed orange), and dark water-only (dashed blue) microcosms. Microcosm concentrations are from the end of the experiment at 36 DAT and error bars show ± standard deviation.

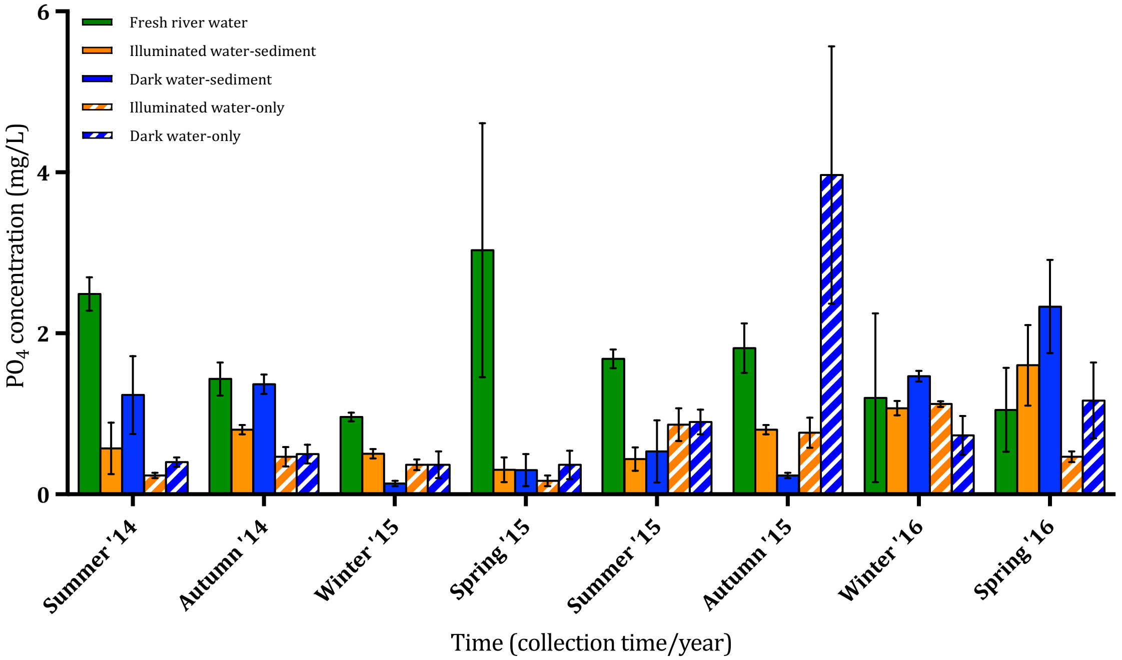

Figure 8. Water PO_4 concentration of the fresh samples and at the end of each collection time. PO_4 concentration in the water was quantified for the fresh samples from the river (solid green) and in the illuminated water-sediment (solid orange), dark water-sediment (solid blue), illuminated water-only (dashed orange), and dark water-only (dashed blue) microcosms. Microcosm concentrations are from the end of the experiment at 36 DAT and error bars show ± standard deviation.

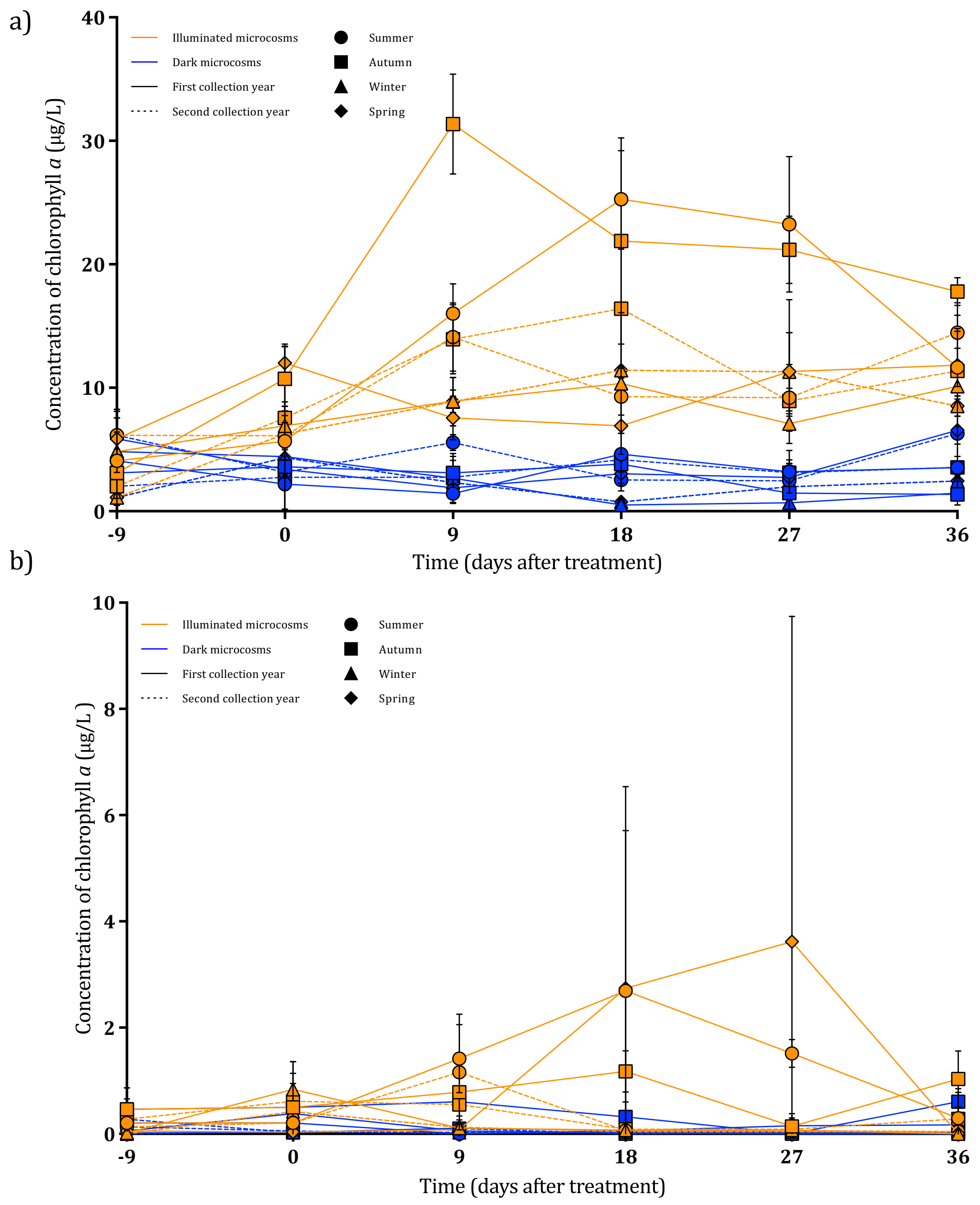

Figure 9. Concentration of chlorophyll *a* in the water-sediment (a) and water-only (b) microcosms. Chlorophyll *a* was extracted from the systems using acetone in illuminated (orange) and dark (blue) microcosms over 36 days in summer (circles), autumn (squares), winter (triangles), and spring (diamonds). The first year of each collection time was denoted by a solid

line and the second year by a dashed line. In the water-sediment systems, the concentrations in the water and the sediment were summed together. Error bars show ± standard deviation.

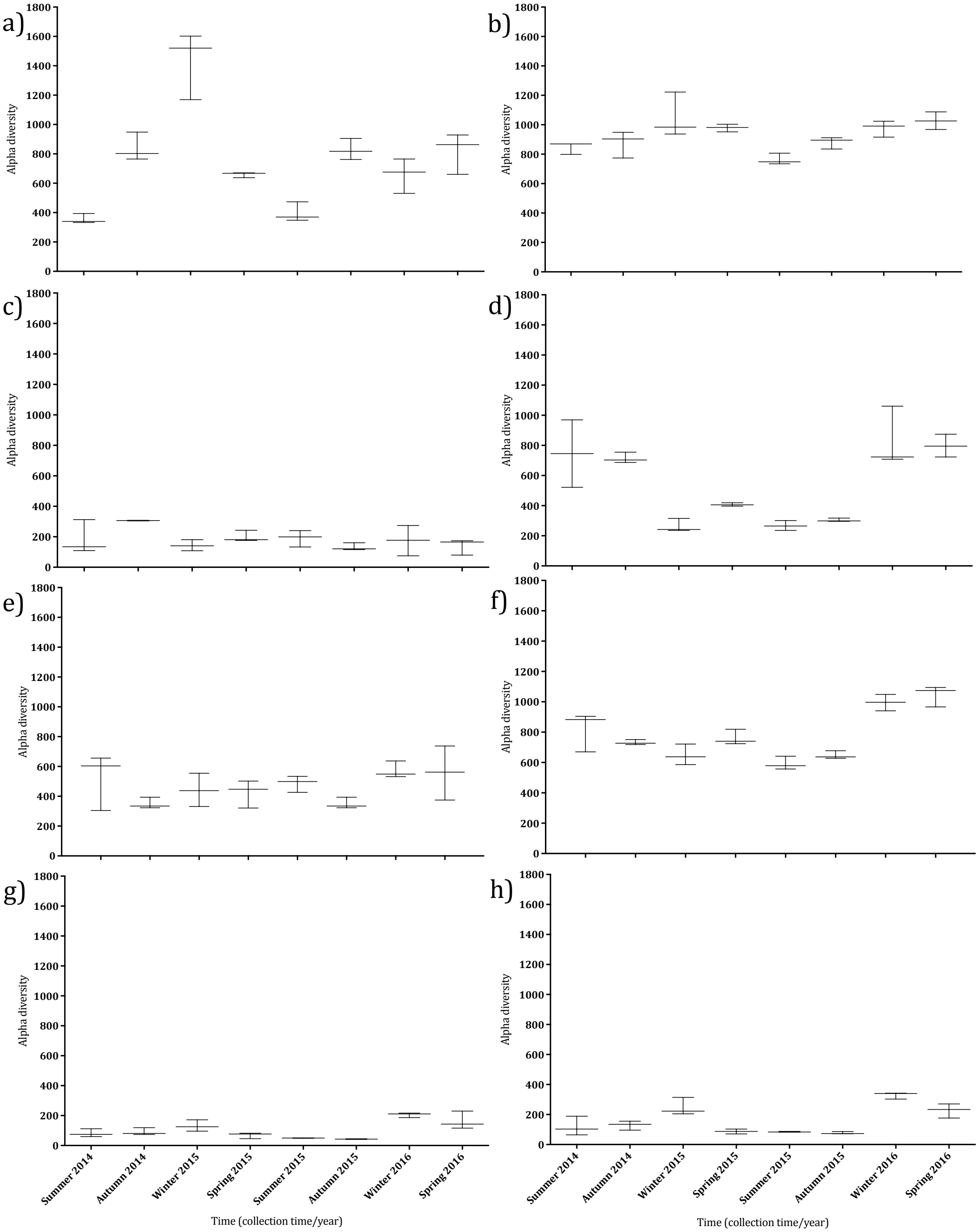

Figure 10. Alpha diversity of bacterial communities between collection times at the sample site and at 36 DAT. Fisher's alpha index was calculated from the observed bacterial species in fresh water (a) and sediment (b), water in illuminated (c) and dark (d) water-sediment microcosms, sediment in illuminated (e) and dark (f) water-sediment microcosms, and water in

illuminated (g) and dark (h) water-only microcosms at 36 DAT. Whiskers show minimum and maximum values and middle lines the median values.

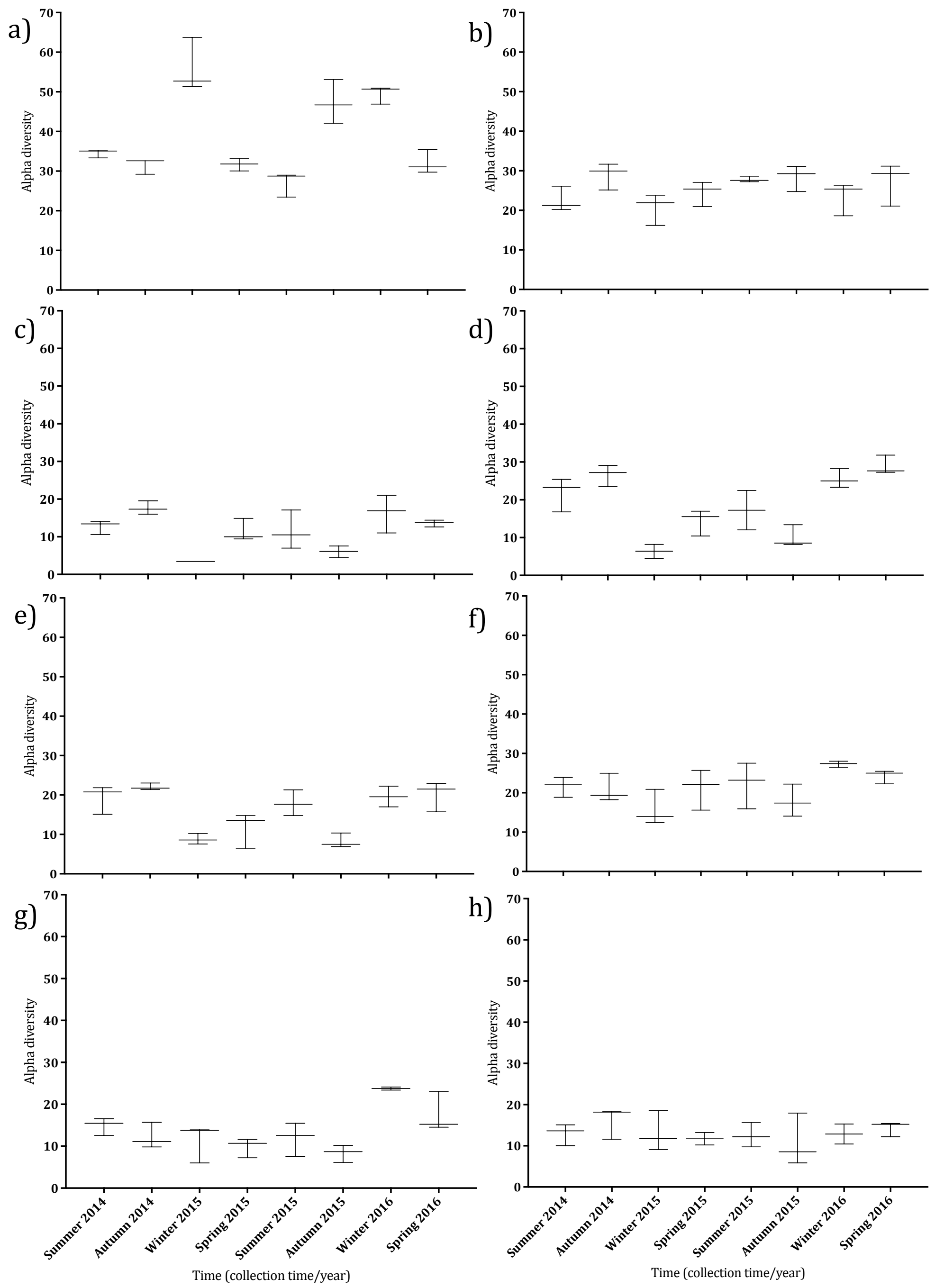

Figure 11. Alpha diversity of phototrophic communities between collection times at the sample site and at 36 DAT. Fisher's alpha index was calculated from the observed bacterial species in fresh water (a) and sediment (b), water in illuminated (c) and dark (d) water-sediment microcosms, sediment in illuminated (e) and dark (f) water-sediment microcosms, and water in

illuminated (g) and dark (h) water-only microcosms at 36 DAT. Whiskers show minimum and maximum values and middle lines the median values.

Tables

Collection time/year	Descriptive statistic	Silt (%)	Clay (%)	Sand (%)	Organic carbon (%)	pH
Summer '14	Mean	10.5	5.4	84.0	1.3	7.6
	SD	3.9	2.4	6.3	0.1	0.1
Autumn '14	Mean	7.9	3.8	88.3	1.2	7.7
	SD	3.2	2.0	5.1	0.3	0.1
Winter '15	Mean	13.7	7.0	79.3	1.3	7.8
	SD	2.5	1.4	3.9	0.1	0.1
Spring '15	Mean	13.6	6.6	79.8	1.0	7.7
	SD	3.3	1.5	4.7	0.1	0.1
Summer '15	Mean	6.3	2.8	90.8	1.7	7.8
	SD	0.5	0.2	0.6	0.1	0.2
Autumn '15	Mean	7.8	4.3	87.9	1.3	7.9
	SD	2.2	1.3	3.5	0.4	0.2
Winter '16	Mean	12.2	6.1	81.7	1.3	7.9
	SD	7.8	4.4	12.2	0.3	0.2
Spring '16	Mean	18.0	9.2	72.8	1.6	8.0
	SD	7.0	3.5	10.6	0.2	0.0

Table 1. Sediment characteristics from the sample site across different collection times. The mean and standard deviation of the sample site sediment characteristics across different collection times. Analysis included silt, clay, and sand content, organic carbon content, and pH and was carried out by Lancrop Laboratories, York, United Kingdom. *SD* denotes *standard deviation*.

Isopyrazam physical-chemical properties	
Chemical formula	$C_{20}H_{23}F_2N_3O$
Molecular weight	359.40 g/mol
Melting point	137 °C
Boiling point	257 °C
Water solubility (25 °C, pH 7)	0.55 mg/L
Log K_{ow} (20 °C, pH 7)	4.25
K_{oc}	1732 – 2491 mL/g
Vapour pressure (20 °C)	2.2×10^{-8} Pa
Henry's Law constant	3.7×10^{-5} Pa m^3/mol
pK_a	No dissociation

Table 2. Physical-chemical properties of isopyrazam. Data taken from PPDB (2017), Abad-Fuentes et al. (2015), and Dæhli et al. (2012).

Time in run (minutes)	0.2 % glacial acetic acid (%)	Acetonitrile (%)
0	90	10
5	90	10
25	30	70
27	10	90
30	10	90
32	90	10
35	90	10

Table 3. HPLC elution gradient of mobile phases for isopyrazam analysis. Mobile phases were 0.2 % glacial acetic acid (Fischer Scientific, UK) and acetonitrile (HPLC grade, Fischer Scientific, UK). A flow rate of 1 mL/minute was used with a ratio of mobile phase to scintillation fluid of 1:1. The column oven temperature was at 20 °C and the radiodetector had a dwell time of 6 seconds.

Sample (collection time/year/microcosm)	DegT50 (days)	k_1	Lower 95 % CI	Upper 95 % CI
Summer '14 illuminated water-sediment	11.1	0.064	0.051	0.074
Summer '14 dark water-sediment	116	0.006	0.003	0.009
Summer '14 illuminated water-only	57.8	0.012	0.007	0.017
Summer '14 dark water-only	101.0	0.007	0.001	0.013
Autumn '14 illuminated water-sediment	9.0	0.077	0.062	0.092
Autumn '14 dark water-sediment	117.0	0.004	0.001	0.007
Autumn '14 illuminated water-only	72.7	0.010	0.006	0.013
Autumn '14 dark water-only	211.0	0.003	0.001	0.006
Winter '15 illuminated water-sediment	64.4	0.011	0.007	0.015
Winter '15 dark water-sediment	203.0	0.003	0.002	0.005
Winter '15 illuminated water-only	139.0	0.005	0.003	0.007
Winter '15 dark water-only	714.0	0.001	-7.7×10^{-5}	0.002
Spring '15 illuminated water-sediment	27.7	0.025	0.018	0.033
Spring '15 dark water-sediment	251.0	0.003	0.002	0.005
Spring '15 illuminated water-only	126.0	0.006	0.002	0.009
Spring '15 dark water-only	278.0	0.002	0.001	0.004
Summer '15 illuminated water-sediment	14.1	0.049	0.037	0.062
Summer '15 dark water-sediment	334.0	0.002	4.7×10^{-4}	0.004
Summer '15 illuminated water-only	115.0	0.006	0.003	0.009
Summer '15 dark water-only	2960.0	2.4×10^{-4}	-0.002	0.002
Autumn '15 illuminated water-sediment	20.0	0.035	0.020	0.049
Autumn '15 dark water-sediment	215.0	0.003	0.002	0.004
Autumn '15 illuminated water-only	58.3	0.012	0.007	0.017
Autumn '15 dark water-only	316.0	0.002	-5.5×10^{-5}	0.004
Winter '16 illuminated water-sediment	25.3	0.027	0.024	0.031
Winter '16 dark water-sediment	170.0	0.004	0.002	0.006
Winter '16 illuminated water-only	134.0	0.005	0.003	0.007
Winter '16 dark water-only	335.0	0.002	0.001	0.004
Spring '16 illuminated water-sediment	17.4	0.040	0.030	0.050
Spring '16 dark water-sediment	195.0	0.004	0.001	0.006
Spring '16 illuminated water-only	43.9	0.016	0.012	0.019
Spring '16 dark water-only	236.0	0.003	0.001	0.005

Table 4. DegT50 and rate constant estimates from Computer Assisted Kinetic Evaluation for microcosm treatments at each collection time. Single First-Order kinetic models were used for all data and 95 % confidence intervals calculated for the rate constant. k_l denotes the first-order kinetics rate constant and *CI* denotes *confidence interval*.

Sample (collection time/year/microcosm)	Model	χ^2 (%)	r^2	Prob. > t (k_1)
Summer '14 illuminated water-sediment	SFO	8.9	0.950	1.3×10^{-8}
Summer '14 dark water-sediment	SFO	3.2	0.580 *	4.5×10^{-4}
Summer '14 illuminated water-only	SFO	5.6	0.647 *	1.5×10^{-4}
Summer '14 dark water-only	SFO	4.4	0.318 *	1.5×10^{-2}
Autumn '14 illuminated water-sediment	SFO	12.7	0.954	2.6×10^{-8}
Autumn '14 dark water-sediment	SFO	4.1	0.420 *	4.4×10^{-3}
Autumn '14 illuminated water-only	SFO	3.4	0.693 *	5.5×10^{-5}
Autumn '14 dark water-only	SFO	3.7	0.393 *	5.8×10^{-5}
Winter '15 illuminated water-sediment	SFO	3.2	0.742	2.0×10^{-5}
Winter '15 dark water-sediment	SFO	2.2	0.544 *	8.0×10^{-4}
Winter '15 illuminated water-only	SFO	1.5	0.627 *	2.2×10^{-4}
Winter '15 dark water-only	SFO	0.8	0.234 *	3.3×10^{-2}
Spring '15 illuminated water-sediment	SFO	10.6	0.8066	3.3×10^{-6}
Spring '15 dark water-sediment	SFO	2.3	0.467 *	2.4×10^{-3}
Spring '15 illuminated water-only	SFO	4.7	0.444 *	2.9×10^{-3}
Spring '15 dark water-only	SFO	1.0	0.469 *	2.4×10^{-3}
Summer '15 illuminated water-sediment	SFO	13	0.881	5.1×10^{-7}
Summer '15 dark water-sediment	SFO	1.8	0.374 *	7.6×10^{-3}
Summer '15 illuminated water-only	SFO	2.4	0.639 *	1.8×10^{-4}
Summer '15 dark water-only	SFO	2.0	0.004 *	4.1×10^{-1} *
Autumn '15 illuminated water-sediment	SFO	19.4 *	0.689 *	9.5×10^{-5}
Autumn '15 dark water-sediment	SFO	0.9	0.809	2.5×10^{-6}
Autumn '15 illuminated water-only	SFO	4.8	0.673 *	1.3×10^{-4}
Autumn '15 dark water-only	SFO	2.2	0.251 *	2.8×10^{-2}
Winter '16 illuminated water-sediment	SFO	4.4	0.953	5.4×10^{-10}
Winter '16 dark water-sediment	SFO	1.7	0.545 *	8.7×10^{-4}
Winter '16 illuminated water-only	SFO	1.1	0.699 *	5.2×10^{-5}
Winter '16 dark water-only	SFO	1.7	0.424 *	4.5×10^{-3}
Spring '16 illuminated water-sediment	SFO	12.5	0.875	4.8×10^{-7}
Spring '16 dark water-sediment	SFO	3.1	0.447 *	2.9×10^{-3}
Spring '16 illuminated water-only	SFO	1.3	0.894	6.8×10^{-8}
Spring '16 dark water-only	SFO	1.4	0.551 *	7.6×10^{-4}

Table 5. Kinetic model and acceptance requirements for DegT50 and rate constant estimates from Computer Assisted Kinetics Evaluation for microcosm treatments at each collection time. SFO kinetic models were used for all data and key acceptance requirements are goodness of fit (χ^2), correlation between the observed and expected values (r^2), and the probability that the rate constant was significantly different to zero (Prob. > t). * denotes values

that have failed the acceptance requirements and k_1 denotes the first-order kinetics rate constant. Acceptance requirements are as follows: goodness of fit ($\chi^2 < 15$ %), assessment of whether the degradation rate constant differs from zero (t-test, probability ≤ 0.05), and correlation between the observed and the expected values ($r^2 \geq 0.7$).

Methods

Method 1. DNA isolation and quantification.

Fresh water and sediment taken from the sample site and water and sediment from the microcosms at 36 DAT were analysed. Water samples were filtered on Whatman 0.2 μm pore size and 47 mm diameter Anodisc filters (GE Healthcare, UK) and DNA was isolated from both the water filters and 0.5 g sediment using FastDNA™ SPIN Kit for Soil (MP Biomedicals, US) and the FastPrep® Instrument (MP Biomedicals, US) according to the manufacturer's protocol. The quantity of the DNA extracts were analysed using the Qubit® 2.0 (Invitrogen, US) high sensitivity fluorometric quantitation protocol according to the manufacture's guidelines and extracts were normalised to 1 ng/mL by dilution with Just Water double distilled molecular biology grade water (Microzone Limited, UK). Primers were designed so that Nextera XT transposase sequences were added to the published amplicon primer sequences. Bacterial diversity was analysed using primer sets as in Caporaso et al. (2011) and phototrophic diversity was analysed using primer sets as in Sherwood and Presting (2007). PCR was performed for each extract using 13 μL Q5® Hot Start High-Fidelity DNA polymerase 2x Master Mix (New England Biolabs, US), 10 μL DNA (1 μg/mL), 0.4 μL BSA (Sigma-Aldrich, US), and 1.3 μL of both the forward and reverse primers (10 μM). Samples were run on a GeneAmp PCR System 9700 (Applied Biosystems, US). For 16S rRNA samples, thermocycling consisted of an initial denaturation at 98 °C for 30 seconds, followed by 25 cycles of 98 °C for 10 seconds, 50 °C for 15 seconds, and 72 °C for 20 seconds. The final extension was at 72 °C for 5 minutes. For 23S rRNA samples, thermocycling consisted of an initial denaturation at 94 °C for 20 seconds, followed by 25 cycles of 94 °C for 20 seconds, 55 °C for 30 seconds, and 72 °C for 30 seconds. The final extension was at 72 °C for 10 minutes.

The PCR products were purified using Agencourt AMPure XP beads (Beckman Coulter, UK) according to the manufacture's protocol. The adapted amplicons were modified by attaching indices and Illumina sequencing adapters using the Nextera XT Index Kit v2 (Illumina Inc., US) by PCR as described in the manufacturer's protocol. Samples were normalised using the SequalPrep™ Normalisation Plate Kit (Invitrogen, US) according to the manufacturer's protocol and quantitatively assessed using Qubit® 2.0 (Invitrogen, US) as above. The final pooled library concentration was 4 nM and this was sequenced using the MiSeq Reagent Kit v3 600-cycle (Illumina, US).

Raw sequences were automatically de-multiplexed by the Illumina MiSeq. Trimmomatic version 0.35 (Bolger et al., 2014) was used to remove any low-quality bases from sequence ends. USEARCH and UPARSE software (Edgar, 2010, Edgar, 2013) were used as in Orchard et al. (2017) and returned sequences were clustered to operational taxonomic units (OTUs) at 97 % minimum identify threshold using the -cluster_otus command, which also filters chimeras. For additional chimera filtering, the GOLD database (Edgar et al., 2011) was used for 16S rRNA genes and de novo chimera checks in the UPARSE pipeline were used for 23S rRNA genes. QIIME version 1.8 (Caporaso et al., 2010), as well as the Greengenes reference database (McDonald et al., 2012) for 16S rRNA sequences and the ARB SILVA 119 LSU Ref database (Quast et al., 2013) for 23S rRNA sequences, were used to assign taxonomies. For 16S reads, only bacterial sequences were retained and any of mitochondrial or chloroplast origin were removed. For 23S rRNA reads, many OTUs were unassigned and so taxonomy assignment was carried out by alignment in ARB (Ludwig et al., 2004) based on the position of the sequence in the tree. Only phototrophic taxa were retained and any eukaryotic samples assigned as "Metazoa" were removed. All samples were rarefied to an even sampling depth and this was

determined ad-hoc to ensure that an acceptable number of sequences were maintained, yet the maximum number of samples possible were included.

References

Abad-Fuentes, A., E. Ceballos-Alcantarilla, J. V. Mercader, C. Agulló, A. Abad-Somovilla and F. A. Esteve-Turrillas (2015). "Determination of succinate-dehydrogenase-inhibitor fungicide residues in fruits and vegetables by liquid chromaography-tandem mass spectometry - electronic supplementary material." Analytical and Bioanalytical Chemistry **407**(14): 4207-4211.

Bolger, A. M., M. Lohse and B. Usadel (2014). "Trimmomatic: a flexible trimmer for Illumina sequence data." Bioinformatics **30**(15): 2114-2120.

Caporaso, J. G., J. Kuczynski, J. Stombaugh, K. Bittinger, F. D. Bushman, E. K. Costello, N. Fierer, A. G. Peña, J. K. Goodrich, J. I. Gordon, G. A. Huttley, S. T. Kelley, D. Knights, J. E. Koenig, R. E. Ley, C. A. Lozupone, D. McDonald, B. D. Muegge, M. Pirrung, J. Reeder, J. R. Sevinsky, P. J. Turnbaugh, W. A. Walters, J. Widmann, T. Yatsunenko, J. Zaneveld and R. Knight (2010). "QIIME allows analysis of high-throughput community sequencing data." Nature Methods **7**: 335-336.

Caporaso, J. G., C. L. Lauber, W. A. Walters, D. Berg-Lyons, C. A. Lozupone, P. J. Turnbaugh, N. Fierer and R. Knight (2011). "Global patterns of 16S rRNA diversity at a depth of millions of sequences per sample." Proceedings of the National Academy of Sciences **108**(1): 4516-4522.

Dæhli, M., M. Randall and R. Holten (2012). "Evaluation of the plant protection product Bontima - cyprodinil + isopyrazam regarding application for authorisation." Norwegian Scientific Committee for Food Safety: 1-51.

Edgar, R. C. (2010). "Search and clustering orders of magnitude faster than BLAST." Bioinformatics **26**(19): 2460-2461.

Edgar, R. C. (2013). "UPARSE: highly accurate OTU sequences from microbial amplicon reads." Nature Methods **10**(10): 996-998.

Edgar, R. C., B. J. Hass, J. C. Clemente, C. Quine and R. Knight (2011). "UCHIME improves sensitivity and speed of chimera detection." Bioinformatics **27**(16): 2194-2200.

Google. (2016). "Map data." Retrieved 7th January, 2016, from https://www.google.co.uk/maps/@52.2064492,-1.6073383,14z.

Ludwig, W., O. Strunk, R. Westram, L. Richter, H. Meier, Yadhukumar, A. Buchner, T. Lai, S. Steppi, G. Jobb, W. Forster, I. Brettske, S. Gerber, A. W. Ginhart, O. Gross, S. Grumann, S. Hermann, R. Jost, A. Konig, T. Liss, R. Lussmann, M. May, B. Nonhoff, B. Reichel, R. Strehlow, A. Stamatakis, N. Stuckmann, A. Vilbig, M. Lenke, T. Ludwig, A. Bode and K. H. Schleifer (2004). "ARB: a software environment for sequence data." Nucleic Acids Research **32**: 1363-1371.

McDonald, D., M. N. Price, J. Goodrich, E. P. Nawrocki, T. Z. DeSantis, A. Probst, G. L. Andersen, R. Knight and P. Hugenholtz (2012). "An improved Greengenes taxonomy with explicit ranks for ecological and evolutionary analyses of bacteria and archaea." Multidisciplinary Journal of Microbial Ecology **6**(3): 610-618.

Orchard, S., S. Hilton, G. D. Bending, I. A. Dickie, R. J. Standish, D. B. Gleeson, R. P. Jeffery, J. R. Powell, C. Walker, D. Bass, J. Monk, A. Simonin and M. H. Ryan (2017). "Fine endophytes (*Glomus tenue*) are related to Mucoromycotina, not Glomeromycota." New Phytologist **213**(2): 481-486.

PPDB. (2017). "General Information for Isopyrazam." Pesticide Properties DataBase Retrieved 19/09/17, 2017, from http://sitem.herts.ac.uk/aeru/ppdb/en/Reports/1449.htm.

Quast, C., E. Pruesse, P. Yilmaz, J. Gerken, T. Schweer, P. Yarza, J. Peplies and F. O. Glöckner (2013). "The SILVA ribosomal RNA gene database project: improved data processing and web-based tools." Nucleic Acids Research **41**: 590-596.

Sherwood, A. R. and G. G. Presting (2007). "Universal primers amplify a 23S rDNA plastid marker in eukaryotic algae and cyanobacteria." Journal of Phycology **43**: 605-608.
